# Derivation and Validation of a Prediction Model of End-Stage Renal Disease in Patients With Type 2 Diabetes Based on a Systematic Review and Meta-analysis

**DOI:** 10.3389/fendo.2022.825950

**Published:** 2022-03-10

**Authors:** Qiuyue Ren, Dong Chen, Xinbang Liu, Ronglu Yang, Lisha Yuan, Min Ding, Ning Zhang

**Affiliations:** ^1^ Department of Nephropathy, Wang Jing Hospital of China Academy of Chinese Medical Sciences, Beijing, China; ^2^ Graduate School, Tianjin University of Traditional Chinese Medicine, Tianjin, China; ^3^ NHC Key Laboratory of Hormones and Development, Tianjin Key Laboratory of Metabolic Diseases, Chu Hsien-I Memorial Hospital & Tianjin Institute of Endocrinology, Tianjin Medical University, Tianjin, China; ^4^ Graduate School, Beijing University of Chinese Medicine, Beijing, China

**Keywords:** type 2 diabetes, end-stage renal disease, prediction model, meta-analysis, cohort study

## Abstract

**Objectives:**

To develop and validate a model for predicting the risk of end-stage renal disease (ESRD) in patients with type 2 diabetes.

**Methods:**

The derivation cohort was from a meta-analysis. Statistically significant risk factors were extracted and combined to the corresponding risk ratio (RR) to establish a risk assessment model for ESRD in type 2 diabetes. All risk factors were scored according to their weightings to establish the prediction model. Model performance is evaluated using external validation cohorts. The outcome was the occurrence of ESRD defined as eGFR<15 ml min^-1^ 1.73 m^-2^ or received kidney replacement therapy (dialysis or transplantation).

**Results:**

A total of 1,167,317 patients with type 2 diabetes were included in our meta-analysis, with a cumulative incidence of approximately 1.1%. The final risk factors of the prediction model included age, sex, smoking, diabetes mellitus (DM) duration, systolic blood pressure (SBP), hemoglobin A1c (HbA1c), estimated glomerular filtration rate (eGFR), and triglyceride (TG). All risk factors were scored according to their weightings, with the highest score being 36.5. External verification showed that the model has good discrimination, AUC=0.807(95%CI 0.753–0.861). The best cutoff value is 16 points, with the sensitivity and specificity given by 85.33% and 60.45%, respectively.

**Conclusion:**

The study established a simple risk assessment model including 8 routinely available clinical parameters for predicting the risk of ESRD in type 2 diabetes.

## Introduction

Diabetes has become a serious disease that endangers human health. According to the statistics of the International Diabetes Federation (IDF) in 2017, there are about 463 million adults with diabetes in the world, and the number of diabetes patients is expected to increase to 700 million by 2045 (1). The global increase in patients with diabetes has contributed to the ever-increasing end-stage renal disease (ESRD) prevalence (2). The global percentage of incident ESRD patients due to diabetes increased from 22.1% in 2000 to 31.3% in 2015 (3). Diabetes has become a vital risk factor for ESRD. The combination of diabetes and ESRD leads to not only a lower survival rate (4) but also intense economic burden to patients and the social medical system (5).

Reducing the occurrence and progression of diabetic kidney disease (DKD) is an effective strategy currently (6, 7). Predicting and identifying patients with diabetes at risk for ESRD will contribute to guide treatment, surveillance, and referral strategies. Previously established prediction models of kidney disease were mostly for patients with chronic kidney disease (CKD) (8–10) or DKD (11). The prediction model published by the ADVANCE clinical trial is based on a large prospective cohort study, which is accurate in the risk assessment of disease onset (12), but required a lot of time, manpower, and material resources. Elley et al. developed a 5-year risk of ESRD events model, but the operation method is so complicated that it is not conducive to clinical application (13). Therefore, we aim to establish and verify a stable and reliable model that can be easily applied in clinical practice and public health to predict the risk of ESRD in patients with type 2 diabetes.

## Method

### Study Registration

The review was registered in the International Prospective Register of Systematic Reviews (PROSPERO), registration number: CRD42021279538.

### Study Population

#### Derivation Cohort

**Figure 1:**
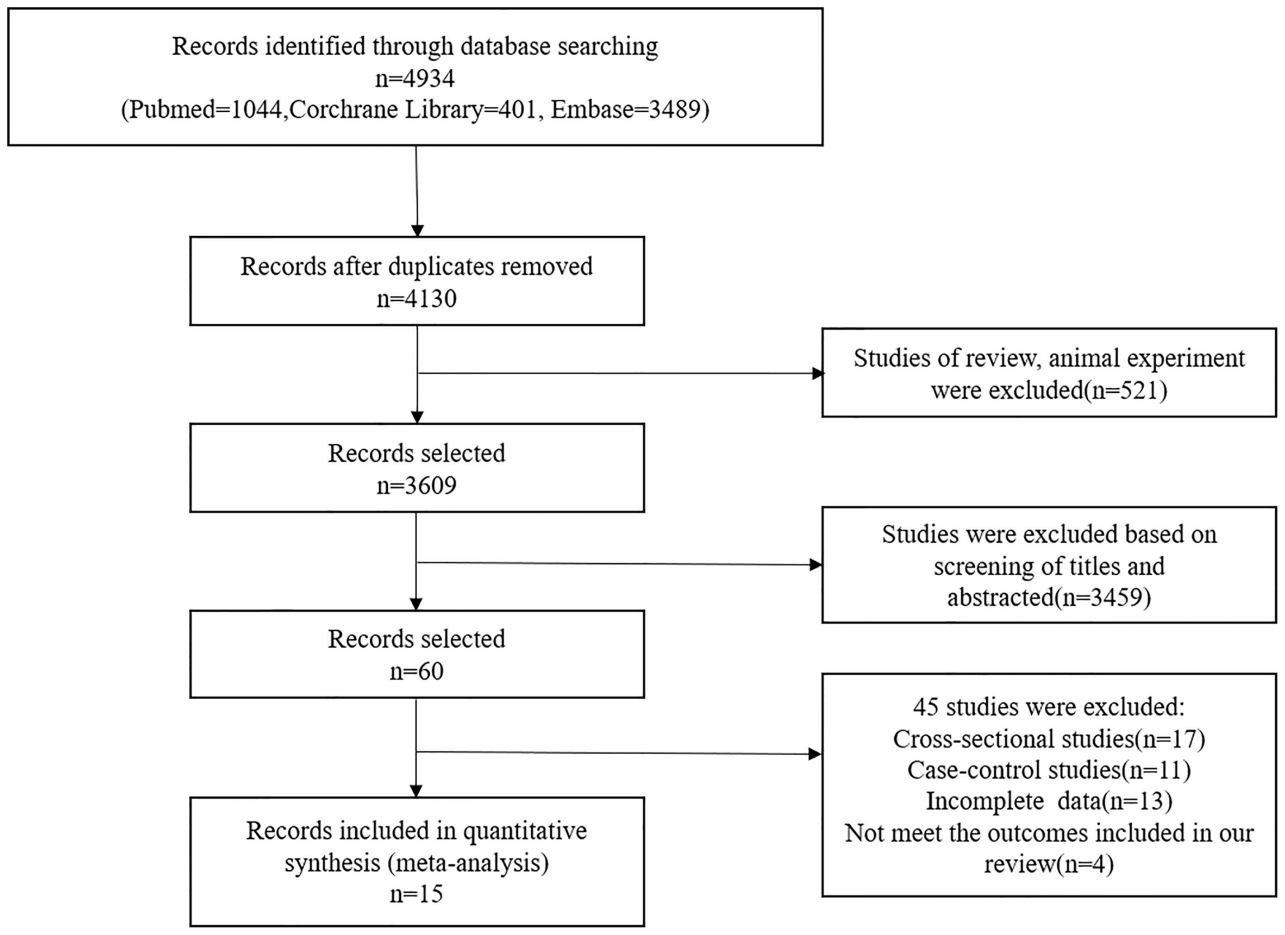
Flow diagram of the literature selection process.

The derivation cohort was based on a systematic review and meta-analysis of 15 cohort studies (14–28), of which 12 were prospective and 3 were retrospective. Electronic databases were retrieved from Pubmed, Corchrane Library, and Embase from its inception to July 2021. The combined text and MeSH heading search strategies were “diabetes mellitus,” “End stage renal disease,” “risk factor,” and “cohort study.” A total of 1,167,317 type 2 diabetes from Asia (China, India), Europe (UK, Italy, France, Finland), and North America (US, Canada) were included in our derivation cohort. These patients had eGFR>15 ml min^-1^ 1.73 m^-2^ [using CKD–Epidemiology Collaboration [CKD–EPI] (29)] and did not receive dialysis or kidney transplantation. In the cohort, 45% were white and 55% were Asian. All included cohort studies had complete baseline data and were assessed using the Newcastle-Ottawa Scale (NOS) (30). The flowchart of studies screening is shown in **Figure 1**. The specific retrieval strategy, inclusion criteria, data extraction, and quality assessment are provided in **Supplementary Data**.

#### Validation Cohort

**Figure 2:**
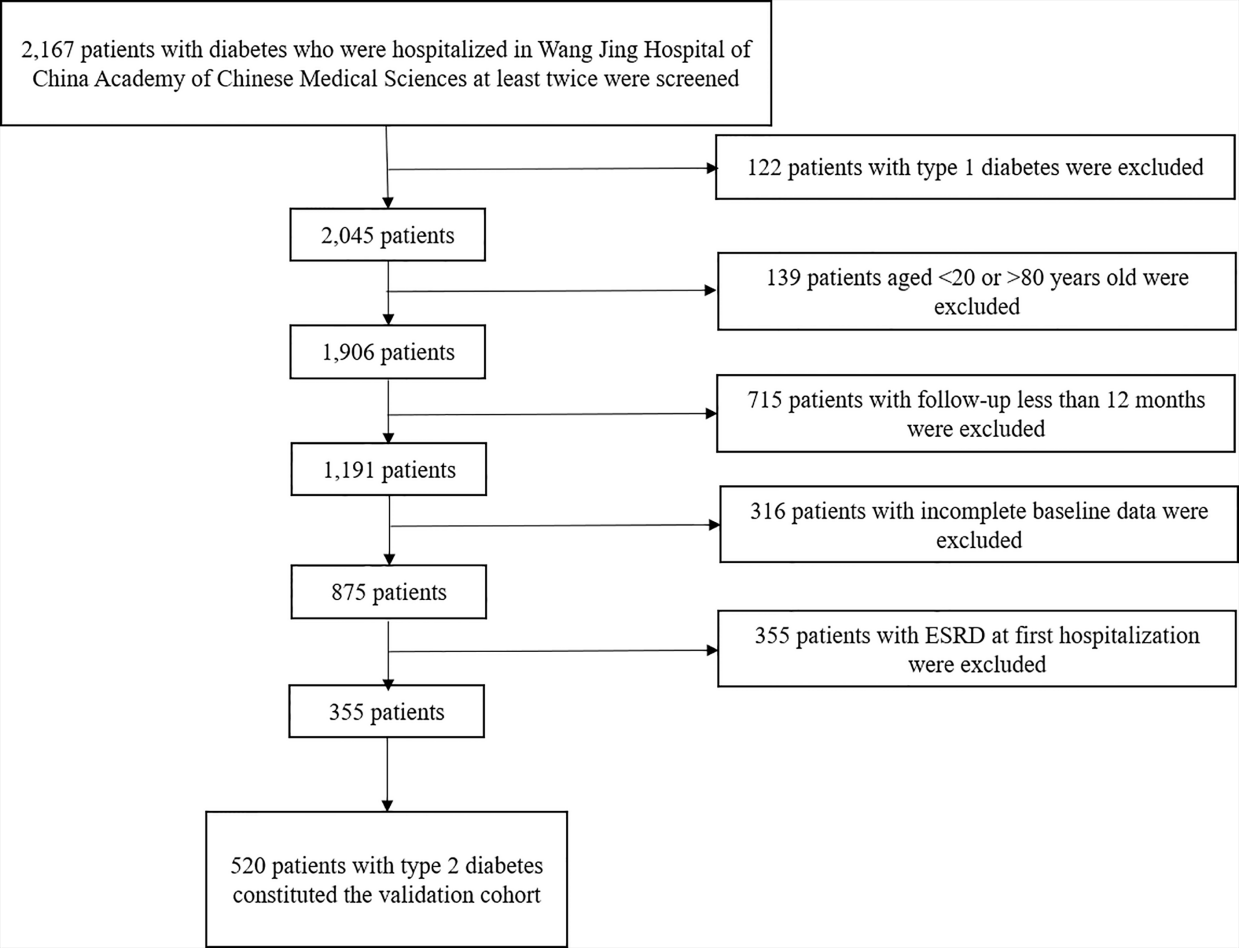
Process for the selection of patients in the validation cohort.

The validation cohort was from type 2 diabetes patients from Wang Jing Hospital of China Academy of Chinese Medical Sciences, who have been hospitalized at least twice from June 2016 to June 2021, between 20 and 80 years old, with eGFR>15 ml min^-1^ 1.73 m^-2^ at initial hospitalization, and progressed to ESRD or initiation of renal replacement therapy (dialysis or kidney transplantation) at last hospitalization. We excluded patients hospitalized for azotemia, acute kidney injury, primary glomerulonephritis, nephrotic syndrome, acute complications of diabetes, and severe cardiovascular and cerebrovascular disease. A total of 2,167 patients with diabetes who were hospitalized at least twice were included in our study; 122 patients with type 1 diabetes, 139 patients younger than 20 years and older than 80 years, 715 patients with follow-up less than 12 months, 316 patients with incomplete baseline data, and 355 patients with ESRD at first hospitalization were excluded. Ultimately, 520 patients with type 2 diabetes were included in the validation cohort. The flowchart is shown in **Figure 2**.

### Outcome

The outcome was the occurrence of ESRD defined as eGFR<15 ml min^-1^ 1.73 m^-2^ or received kidney replacement therapy (dialysis or transplantation) (31).

### Model Development

(1) According to the above systematic review and meta-analysis, all risk factors in the prediction model were selected. We selected appropriate RR and 95% confidence interval by subgroup analysis or sensitivity analysis, then calculate the corresponding β-coefficient. (2) The score was calculated by multiplying the β-coefficient by 10 and rounding it to the best whole number (32). (3) All risk factors in the prediction model were classified and assigned scores to establish a risk score system according to meta-analysis and clinical practice guidelines. The scores of each risk factor were summarized to calculate the total score (33). The higher the cumulative score, the higher the risk of ESRD.

#### Model Validation

External data from the above retrospective cohort study were used to evaluate and validate our risk prediction model. We used the risk score system to calculate the total score of validation cohort baseline variables. A receiver operating characteristic (ROC) curve was constructed under the total score. Sensitivity, specificity, optimal cutoff, and area under the curve (AUC) were calculated based on the ROC curve. The AUC means prediction performance, with the value ranging from 0.5 to 1.0. The higher the value, the better the prediction accuracy. According to the optimal cutoff point, patients were divided into four risk groups, namely, low, moderate, high, and very high risk. Kaplan–Meier curves were used to calculate the cumulative risk of morbidity in different groups. Statistical analysis was performed using the SPSS 23.0 (IBM Corp., Armonk, NY, USA) and STATA software, version 14.0 (StataCorp, College Station, TX).

### Statistical Analysis

#### Meta-analysis

The risk ratios (RRs) with a 95% confidence interval (95%CI) for each risk factor for ESRD were extracted. Heterogeneity was quantified using the Q test and I^2^ statistics. When the heterogeneity test indicated no significance (P>0.1and I^2^<50%), a fixed-effects model was adopted; otherwise, the random effect model was applied. Subgroup analyses were conducted according to the magnitude of the change in the continuous variables. Continuous variables included age (1-year increment), diabetes duration (1-year increment), and hypertension (SBP 1-mmHg increment, DBP 1-mmHg increment). The statistical analysis of the data was performed using RevMan (version 5.3.3; The Cochrane Collaboration) and STATA software, version 14.0 (StataCorp, College Station, TX). *P*-value<0.05 was considered statistically significant unless otherwise specified.

## Results

### Description of the Cohorts

#### Derivation Cohort

We roughly analyzed the baseline of participants enrolled in the cohort study. A total of 1,167,317 diabetes patients were included in the derivation cohort, with age between 20 and 80 years, and 52.5% patients were male. Their diabetes duration ranged from 2 to 24 years. The follow-up was 2.8 to 25 years, equivalent to 3,268,488–29,182,925 person-years. During the follow-up period, 13,187 patients developed ESRD, with incidence 1.1% approximately. Among the cohort, 46%–66% of the patients received insulin therapy, 65%–89% received oral antidiabetic drug (OAD), and 17.9%–24.8% received lipid-lowering treatment. The mean HbA1c ranged from 7.3% to 11.0% (56.3 to 96.7 mmol/mol), the mean BMI ranged from 23.0 to 30 kg/m^2^, the mean SBP ranged from 120 to 160 mmHg, the mean TG ranged from 1.50 to 1.78 mmol/L, and the mean eGFR ranged from 30 to 100 ml min^-1^ 1.73 m^-2^. Baseline characteristics of the derivation cohort are provided in **Supplementary Table 1**. According to the Newcastle-Ottawa scale, all 15 included studies have high quality (provided in **Supplementary Table 2**). These cohort studies had 13 risk factors, including age, sex, body mass index (BMI), smoking, diabetes duration, fasting plasma glucose (FBG), hypertension, HbA1c, albuminuria, eGFR, urine albumin:creatinine ratio (UACR), total cholesterol (TC), and triglyceride (TG). Details of these studies and corresponding cohorts are provided in **Supplementary Table 3**.

#### Validation Cohort

A total of 520 Chinese patients with type 2 diabetes were included in our validation cohort, with 313 males (60.2%) and 207 females (39.8%). The mean age ± SD was 59.2 ± 12.7 years, and the median follow-up was 36 months (interquartile range [IQR]=26–48). At the end of follow-up, 78 patients (57 males and 21 females) had progressed to ESRD. In the baseline data for all patients, diabetes duration (median 10.6 years, [IQR=4.3–17.0]), systolic blood pressure (SBP) (median 132.5 mmHg, [IQR=122.0–144.0]), HbA1c (median 7.8% [61.7 mmol/mol], IQR=7.0 [53]–9.5 [80.3]), eGFR (median 92.1 ml min^-1^ 1.73 m^-2^, [IQR=76.2–103.4]), TG (median 1.53 mmol/L, [IQR=1.0–1.9]), 250 (48.0%) patients were smokers, 235 (45.1%) received OAD, 226 (43.4%) received insulin treatment, 232 (44.6%) received OAD with insulin, 171 (32.9%) patients received angiotensin converting enzyme inhibitors or angiotensin receptor blockers, and 195 (37.5%) patients received statins. The basic information of the validation cohort patients is provided in **Supplementary Table 4**.

### Model Derivation

The 13 risk factors identified in the above systematic review and meta-analysis were included in the ESRD risk assessment model. Reasonably considering heterogeneity and clinical availability, we carefully selected partial subgroup or sensitivity analysis results. Risk factors included in our model were as follows: age incremented by 5–10 years (RR=1.11, 95%CI[1.01, 1.21], *P*=0.02), sex (RR=1.53, 95%CI[1.4, 1.67], *P*<0.001), smoking (RR=1.33, 95%CI[1.15, 1.53], *P*<0.001), DM duration incremented by 1 year (RR=1.02, 95%CI[1.01, 1.03], *P*<0.001), SBP incremented by 1 mmHg (RR=1.01, 95%CI[1.00, 1.01], *P*<0.001), HbA1c incremented by 1% [11 mmol/mol] (RR=1.10, 95%CI[1.08, 1.12], *P*<0.001), eGFR incremented by 1 ml min^-1^ 1.73 m^-2^ (RR=0.97, 95%CI[0.95, 0.99], *P*=0.001), and TG incremented by 1 mmol/L (RR=1.75, 95%CI[1.34, 2.29], *P*<0.001. A forest plot of these factors is shown in **Supplementary Figure 1**; subgroup and sensitivity analyses are shown in **Supplementary Figure 2**. Risk factors, RR (95%CI), β coefficients, and risk scores of risk factors included in the ESRD risk prediction model are shown in **Table 1**.

**Table 1:** Risk stratification, RR (95% CI), β-coefficients, and scores of risk factors included in the ESRD risk prediction model.

Risk factors for ESRD	Pooled RR	95% CI	β-coefficient	Scores
Age (by 5-10 years)	1.11	1.01-1.21	0.10	1.0
Sex (males/females)	1.53	1.4-1.67	0.43	4.0
Smoking (yes/no)	1.33	1.15-1.53	0.29	3.0
DM duration (by 1 year)	1.02	1.01-1.03	0.02	0.2
SBP (by 1 mmHg)	1.01	1.00-1.01	0.01	0.1
HbA1c (by 1% [11 mmol/mol])	1.10	1.08-1.12	0.10	1.0
eGFR (by 1 ml min^-1^ 1.73m^-2^)	0.97	0.95-0.99	-0.03	-0.3
TG (by 1 mmol/L)	1.75	1.34-2.29	0.56	5.5

In conclusion, we developed a simple ESRD risk prediction model: age (years; 20–29 = 0, 30–39 = 1, 40–49 = 2, 50–59 = 3, 60–69 = 4, 70–80 = 5), sex (female=0, male=4), smoking (no=0, yes=3), DM duration (years; <5.0 = 0, 5.0–9.9 = 1, 10.0–14.9 = 2, 15.0–19.9 = 3, ≥20.0 = 4), SBP (mmHg; <130 = 0, 130–139 = 1, 140–149 = 2, ≥150 = 3), HbA1c (<7.0%[<53]=0, 7.0–7.9%[53–63]=1, 8.0–8.9%[64–74]=2, ≥9.0%[≥75]=3), and eGFRmlmin^-1^ 1.73 m^-2^ (≥90 = 0, 60–89 = 3, 45–59 = 4.5, 30–44 = 6, 15–29 = 9). The model, with the maximum score of 36.5, is recommended to be used for type 2 diabetes patients aged 20–80 years who have not progressed to ESRD, mainly white and Asian (detailed in **Table 2**).

**Table 2:** Risk score model of ESRD incident prediction.

Risk factors	Point	Risk factors	Point
Age (years)	DM duration (years)	
20–29	0	<5.0	0
30–39	1	5.0–9.9	1
40–49	2	10.0–14.9	2
50–59	3	15.0–19.9	3
60–69	4	≥20.0	4
70–80	5	**SBP (mmHg)**	
**Sex**		<130	0
Female	0	130–139	1
Male	4	140–149	2
**Smoker** ^#^		≥150	3
No	0	**HbA1c (1% [11mmol/mol])**	
Yes	3	<7.0 [<53]	0
**eGFR** ^##^ **(1 ml min^-1^1.73m^-2^)**		7.0–7.9 [53–63]	1
≥90	0	8.0–8.9 [64–74]	2
60–89	3	≥9.0 [≥75]	3
45–59	4.5	**TG (mmol/L)**	
30–44	6	<1.70	0
15–29	9	≥1.70	5.5

^#^Smoker was defined as having smoked more than 100 cigarettes in their lifetime. ^##^eGFR using CKD–Epidemiology Collaboration [CKD–EPI]. A highest score of risk assessment model is 36.5.

### Model Validation

**Figure 3:**
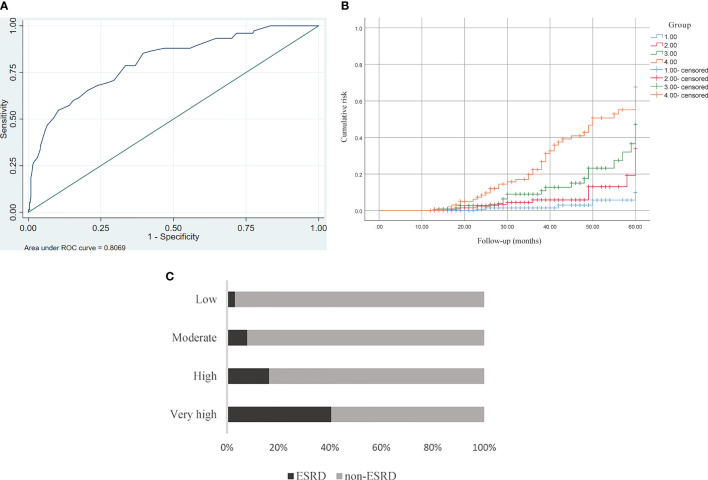
**(A)** ROC curve analysis for predicting ESRD. The AUC was 0.807 (95%CI 0.753–0.861). **(B)** Kaplan–Meier curve of ESRD endpoint for each risk group. Compared with the low-risk group, high-risk group: 5.99, 95%CI (2.17–16.6), P < 0.01, high-risk group: 20.89, 95%CI (7.91–55.18) P < 0.01. **(C)** Prevalence of ESRD in four risk groups stratified by risk score in the validation cohort. Low<12, moderate 12.5–16, high 16–20, very high 20.5–36.5.

The AUC value of the validation cohort was 0.807 (95%CI 0.753–0.861). The ROC curve is showed in **Figure 3A**. The optimal cutoff risk score of ESRD was 16 based on the maximum Youden index, with a sensitivity of 85.33% and specificity of 60.45%. Sensitivity, specificity, and Youden indexes of different critical risk scores are provided in **Supplementary Table 5**. According to the obtained frequencies of the ESRD risk score using different risk scores, 520 patients with type 2 diabetes were divided into 4 groups: low (n=153), moderate (n=128), high (n=116), and very high (n=106) risk. The corresponding risk scores were <12, 12.5–16, 16–20, and 20.5–36.5, respectively. The Kaplan–Meier curve was used to calculate the cumulative risk for each group. Compared with the low-risk group, the RRs of developing ESRD in the high and very high-risk groups were (5.99, 95%CI [2.17–16.6]) and (20.89, 95%CI [7.91–55.18]), respectively (P<0.01) (details are shown in **Figures 3B, C**).

## Discussion

Compared with people without diabetes, those with type 2 diabetes have three to five times the risk of developing ESRD (34). At present, apart from tight glycemic and blood pressure control, and disturbance of lipid metabolism regulation, a few effective interventions can reduce the progression of renal disease in diabetes. Therefore, early detection and treatment are recommended on account of they could reduce the progression of the disease. Our study established a simple and clinically applicable risk prediction model based on a high-quality systematic review and meta-analysis. The model can reliably distinguish between low-risk and high-risk groups. Low-risk patients will be followed by primary healthcare physicians for regular risk assessment without additional inspection and treatment, while high-risk patients will be recommended to receive more intensive test and early intervention.

Based on 15 cohort studies of 1,167,317 patients, the following 8 risk factors were identified: age, sex, smoking, DM duration, SBP, HbA1c, eGFR, and TG. The dominance of sex, smoking, and TG in predicting risk is evident in our meta-analysis. Male patients were more likely to develop to ESRD in our study, which is consistent with many previous studies (8, 24, 35). This may be related to innate sex-related variability in renal structure, gender differences in glomerular hemodynamics, and the direct effects of estrogen and androgens on kidney tissue (36, 37). Smoking increased the risk of ESRD (38), and the risk increased with smoking duration and the number of cigarettes smoked daily (39). In our study, the risk for ESRD increased by 33% in patients with diabetes who smoked. Therefore, quitting or reducing smoking is an essential measure for diabetes patients to prevent ESRD. Dyslipidemia is closely related to renal dysfunction in diabetes patients (40); hyperlipidemia may exacerbate progressive renal disease (41). According to the RENAAL study, triglyceride was significantly associated with the primary outcome (the composite of a doubling of the baseline serum creatinine concentration, end-stage renal disease, or death) in type 2 diabetes and nephropathy (42). Peritoneal dialysis patients with diabetes coexisting with hyperlipidemia have higher all-cause mortality (43). Age and HbA1c are major risk factors for ESRD. In our study, with age incremented by 5–10 years, the risk for ESRD increased by 11%. Several large prospective randomized controlled clinical studies have shown that intensive glycemic control can reduce the occurrence and progression of nephropathy in both type 1 and type 2 diabetes mellitus (44, 45). As HbA1c increased by 1% (11mmol/mol), the risk for ESRD increased by 10%. In addition, our study found that DM duration was also a main risk factor; as DM duration increased by 5 years, the risk for ESRD increased by 10%. Several clinical guidelines and consensus recommend screening for renal disease in patients with type 2 diabetes immediately after diagnosis (31, 46). We found that SBP also played a significant role in predicting ESRD. This also confirmed the results of the correlation between SBP and progression of renal disease shown in other studies (12, 47). According to the CRIC study, there is a 2.6-fold increased risk of the composite renal endpoint for those with mean SBP of 130–139 mmHg compared to <120 mmHg (48). eGFR is a robust predictor for ESRD.As eGFR increased by 5 ml min^-1^ 1.73m^-2^, the risk for ESRD increased by 15% in our study. Several studies have confirmed that apart from eGFR, UACR is also a major factor in predicting the risk of ESRD in diabetic patients (49, 50). However, UACR has a few limitations. The progression of proteinuria is inconsistent with the severity of renal impairment, which showed more obviously in type 2 diabetic patients (51, 52). In our meta-analysis, there was significant heterogeneity (I^2 =^ 100%) in the 7 studies involving UACR, and this heterogeneity could not be reduced or eliminated by subgroup analysis or sensitivity analysis, so we excluded UACR from our prediction model. However, we still cannot ignore the significance of UACR in the progression of kidney disease, and this also provides a direction for our future research.

We established a risk prediction model for ESRD based on systematic reviews and meta-analyses of 15 high-quality cohort studies (including 1,167,317 patients with type 2 diabetes), which greatly enhanced the performance of the model when compared with other *post hoc* analyses or small cross-sectional studies based on randomized controlled trials. We developed a clinical scoring model that includes 8 clinically common variables and does not require complex mathematical calculations, making it easy for clinicians and patients themselves to evaluate risk and initiate timely interventions. In addition, 520 patients with type 2 diabetes were selected as an external cohort to verify our model. The model has good performance with an AUC value of 0.807 (95%CI 0.753–0.861). The best cutoff value is 16 points, with sensitivity and specificity of 85.33% and 60.45%, respectively. We further divided the patients into low-risk, medium-risk, high-risk, and very high-risk groups according to their risk scores. Compared with the low-risk group, those in high- and very high-risk groups had 5.99- and 20.89-fold increases, respectively, in the rate of developing ESRD, further highlighting the importance of our prediction model.

Our analysis has a few limitations. First, the 15 articles we included are inevitably heterogeneous due to diversities in research methods and different race of the included cohorts. Although heterogeneity was reduced by subgroup analysis and sensitivity analysis, the cause for heterogeneity of some indicators was still not explicit. Second, although we included a wide range of type 2 diabetes in our meta-analysis, certain ethnic with increased risk for ESRD such as the black race are underrepresented. And we only validated the model in a retrospective cohort study of diabetes from China, so we need to conduct an external validation in other countries and ethnic groups in the future. Third, some clinical indicators, such as albuminuria, UACR, BMI, and other factors that may affect the progression of kidney disease, were not included in the model due to the heterogeneity or lack of relevant cohort studies. We further need to update our model. In addition, we did not analyze all-cause mortality in our model. Although diabetes complicated with ESRD are at higher risk for mortality, it is more meaningful to learn about the probability of ESRD among survivors.

## Conclusions

In conclusion, we developed and validated a simple prediction model for progression of type 2 diabetes to ESRD based on systematic review and meta-analysis. Our model combines individual lifestyle and routinely obtained laboratory tests, including age, sex, smoking, DM duration, SBP, HbA1c, eGFR, and TG. The model can be widely applied to type 2 diabetes to guide clinical decision.

## Data Availability Statement

The original contributions presented in the study are included in the article/**Supplementary Material**. Further inquiries can be directed to the corresponding author.

## Ethics Statement

Ethical review and approval was not required for the study on human participants in accordance with the local legislation and institutional requirements. Written informed consent for participation was not required for this study in accordance with the national legislation and the institutional requirements.

## Author Contributions

QR, DC, and XL performed the research, analyzed the data, and wrote the manuscript. RY, LY, and MD contributed to collecting the data. NZ contributed to concept and design, study quality assessment, and manuscript revision. All authors contributed to the article and approved the submitted version.

## Funding

This work was supported by the National Natural Science Foundation of China (81973801), Capital Health development Scientific Research project (2018-1-4161), and The Science & Technology Development Fund of Tianjin Education Commission for Higher Education (2018KJ047).

## Conflict of Interest

The authors declare that the research was conducted in the absence of any commercial or financial relationships that could be construed as a potential conflict of interest.

## Publisher’s Note

All claims expressed in this article are solely those of the authors and do not necessarily represent those of their affiliated organizations, or those of the publisher, the editors and the reviewers. Any product that may be evaluated in this article, or claim that may be made by its manufacturer, is not guaranteed or endorsed by the publisher.
