# Supplementary material for: Derivation and Validation of a Prediction Model of End-Stage Renal Disease in Patients With Type 2 Diabetes Based on a Systematic Review and Meta-analysis
Source: Front Endocrinol (Lausanne). 2022 Mar 10;13:825950. doi: 10.3389/fendo.2022.825950 (PMC8960850; doi:10.3389/fendo.2022.825950)
Supplement: Supplementary file 1 [file DataSheet_1.docx]

**SUPPLEMENTARY DATA**

**Systematic review and meta-analysis to identify the risk factors for end-stage renal disease (ESRD) in patients with Type 2 diabetes.**

This study was based on the Preferred Reporting Items for Systematic and Meta-analysis (PRISMA) statement ^[1]^ and the Meta-analysis of Observational Studies in Epidemiology (MOOSE) statement^[2]^.

**Search strategy**

Pubmed, Embase, and Cochrane Library were electronically searched about the risk factors for ESRD from the time of their inception to July 2021 by the following major search terms: “diabetes”, “ESRD”, “risk factors” and “cohort study”. Two independent authors (QY-R and D-C) screened all titles and abstracts to determine independently. The full-texts were browsed when study could not be determined by the abstracts. Any disagreements were resolved through consulting two other senior researchers (N-Z and TX-L) to attain consensus. There was no restriction on language of publication.

***Detailed search strategy in Pubmed***

#1 "Diabetes Mellitus, Type 2"[Mesh]

#2 ((Type 2 Diabetes Mellitus[Title/Abstract]) OR (Type 2 Diabetes，Diabetes[Title/Abstract])) OR (Type 2[Title/Abstract])

#3 #1 OR #2

#4"Kidney Failure, Chronic"[Mesh]

#5(((((((((((((((End-Stage Kidney Disease[Title/Abstract]) OR (Disease, End-Stage Kidney[Title/Abstract])) OR (End Stage Kidney Disease[Title/Abstract])) OR (Kidney Disease, End-Stage[Title/Abstract])) OR (Chronic Kidney Failure[Title/Abstract])) OR (End-Stage Renal Disease[Title/Abstract])) OR (Disease, End-Stage Renal[Title/Abstract])) OR (End Stage Renal Disease[Title/Abstract])) OR (Renal Disease, End-Stage[Title/Abstract])) OR (Renal Disease, End Stage[Title/Abstract])) OR (Renal Failure, End-Stage[Title/Abstract])) OR (End-Stage Renal Failure[Title/Abstract])) OR (Renal Failure, End Stage[Title/Abstract])) OR (Renal Failure, Chronic[Title/Abstract])) OR (Chronic Renal Failure[Title/Abstract])) OR (ESRD[Title/Abstract])

#6 #4 OR #5

#7 ((((((((((((((((((((((("Risk Factors"[Mesh]) OR "Hypertension"[Mesh]) OR "Blood Pressure"[Mesh]) OR "Hyperglycemia"[Mesh]) OR "Hyperuricemia"[Mesh]) OR "Uric Acid"[Mesh]) OR "Lipids"[Mesh]) OR "Triglycerides"[Mesh]) OR "Cholesterol"[Mesh]) OR "Hyperlipidemias"[Mesh]) OR "Obesity"[Mesh]) OR "Gender Identity"[Mesh]) OR "Sex"[Mesh]) OR "Smoking"[Mesh]) OR "Drinking"[Mesh]) OR "Diabetic Retinopathy"[Mesh]) OR "Body Mass Index"[Mesh]) OR "Glycated Hemoglobin A"[Mesh]) OR "Cholesterol, HDL"[Mesh]) OR "Albuminuria"[Mesh]) OR "Proteinuria"[Mesh]) OR "Glomerular Filtration Rate"[Mesh]) OR "Creatinine"[Mesh]) OR "Blood Urea Nitrogen"[Mesh]

#8 (((((((((predicted factor[Title/Abstract]) OR (microalbuminuria[Title/Abstract])) OR (Region[Title/Abstract])) OR (race[Title/Abstract])) OR (family history[Title/Abstract])) OR (age[Title/Abstract])) OR (duration[Title/Abstract])) OR (HbA1c[Title/Abstract])) OR (SBP[Title/Abstract])) OR (DBP[Title/Abstract])

#9 #7 OR #8

#10 ((((((("Cohort Studies"[Mesh]) OR "Case-Control Studies"[Mesh]) OR "Cross-Sectional Studies"[Mesh]) OR "Longitudinal Studies"[Mesh]) OR "Follow-Up Studies"[Mesh]) OR "Prospective Studies"[Mesh]) OR "Retrospective Studies"[Mesh]) OR ((((cohort[Title/Abstract]) OR (longitudinal[Title/Abstract])) OR (prospective[Title/Abstract])) OR (retrospective[Title/Abstract]))

#11 #3 and #6 and #9 and #10

***Detailed search strategy in Embase***

#1 'non insulin dependent diabetes mellitus'/exp

#2 'type 2 diabetes mellitus':ab,ti OR 'type 2 diabetes':ab,ti OR 'diabetes, type 2':ab,ti

#3 #1 OR #2

#4 'chronic kidney failure'/exp

#5 'end-stage kidney disease':ab,ti OR 'disease, end-stage kidney':ab,ti OR 'end stage kidney disease':ab,ti OR 'kidney disease, end-stage':ab,ti OR 'chronic kidney failure':ab,ti OR 'end-stage renal disease':ab,ti OR 'disease, end-stage renal':ab,ti OR 'end stage renal disease':ab,ti OR 'renal disease, end-stage':ab,ti OR 'renal disease, end stage':ab,ti OR 'renal failure, end-stage':ab,ti OR 'end-stage renal failure':ab,ti OR 'renal failure, end stage':ab,ti OR 'renal failure, chronic':ab,ti OR 'chronic renal failure':ab,ti OR 'esrd':ab,ti

#6 #4 OR #5

#7'risk factor'/exp OR 'hypertension'/exp OR 'blood pressure'/exp OR 'hyperglycemia'/exp OR 'uric acid'/exp OR 'lipid'/exp OR 'triacylglycerol'/exp OR 'cholesterol'/exp OR 'hyperlipidemia'/exp OR 'obesity'/exp OR 'gender identity'/exp OR 'sex'/exp OR 'smoking'/exp OR 'drinking'/exp OR 'diabetic retinopathy'/exp OR 'body mass'/exp OR 'hemoglobin a1c'/exp OR 'high density lipoprotein cholesterol'/exp OR 'albuminuria'/exp OR 'proteinuria'/exp OR 'glomerulus filtration rate'/exp OR 'creatinine'/exp OR 'urea nitrogen blood level'/exp

#8 'predicted factor':ab,ti OR 'microalbuminuria':ab,ti OR 'region':ab,ti OR 'race':ab,ti OR 'family history':ab,ti OR 'age':ab,ti OR 'duration':ab,ti OR 'hba1c':ab,ti OR 'sbp':ab,ti OR 'dbp':ab,ti

#9 #7 OR #8

#10'cohort analysis'/exp OR 'case control study'/exp OR 'cross-sectional study'/exp OR 'longitudinal study'/exp OR 'follow up'/exp OR 'prospective study'/exp OR 'retrospective study'/exp

#11#3 AND #6 AND #9 AND #10

***Detailed search strategy in Cochrane Library***

#1 MeSH descriptor: [Diabetes Mellitus, Type 2] explode all trees

#2 (Type 2 Diabetes Mellitus):ti,ab,kw OR (Type 2 Diabetes):ti,ab,kw OR (Diabetes, Type 2):ti,ab,kw

#3 #1 or #2

#4 MeSH descriptor: [Kidney Failure, Chronic] explode all trees

#5 (End-Stage Kidney Disease):ti,ab,kw OR (Disease, End-Stage Kidney):ti,ab,kw OR (End Stage Kidney Disease):ti,ab,kw OR (Kidney Disease, End-Stage):ti,ab,kw OR (Chronic Kidney Failure):ti,ab,kw OR (End-Stage Renal Disease):ti,ab,kw OR (Disease, End-Stage Renal):ti,ab,kw OR (End Stage Renal Disease):ti,ab,kw OR (Renal Disease, End-Stage):ti,ab,kw OR (Renal Disease, End Stage):ti,ab,kw OR (Renal Failure, End-Stage):ti,ab,kw OR (End-Stage Renal Failure):ti,ab,kw OR (Renal Failure, End Stage):ti,ab,kw OR (Renal Failure, Chronic):ti,ab,kw OR (Chronic Renal Failure):ti,ab,kw OR (ESRD ):ti,ab,kw

#6 #4 or #5

#7 MeSH descriptor: [Risk Factors] explode all trees

#8 (hypertension):ti,ab,kw OR (blood pressure):ti,ab,kw OR (hyperglycemia):ti,ab,kw OR (hyperuricemia):ti,ab,kw OR (uric acid ):ti,ab,kw OR (lipids):ti,ab,kw OR (triglycerides):ti,ab,kw OR (cholesterol ):ti,ab,kw OR (hyperlipidemias):ti,ab,kw OR (obesity):ti,ab,kw OR (gender identity):ti,ab,kw OR (sex):ti,ab,kw OR (smoking):ti,ab,kw OR (drinking):ti,ab,kw OR (Diabetic retinopathy):ti,ab,kw OR (body mass index):ti,ab,kw OR (Glycated Hemoglobin A):ti,ab,kw OR (Cholesterol, HDL):ti,ab,kw OR (albuminuria):ti,ab,kw OR (proteinuria):ti,ab,kw OR (glomerular filtration rate):ti,ab,kw OR (Creatinine):ti,ab,kw OR (Blood Urea Nitrogen):ti,ab,kw OR (predicted factor):ti,ab,kw OR (microalbuminuria):ti,ab,kw OR (Region):ti,ab,kw OR (race):ti,ab,kw OR (family history):ti,ab,kw OR (age):ti,ab,kw OR (duration):ti,ab,kw OR (HbA1c):ti,ab,kw OR (SBP ):ti,ab,kw OR (DBP):ti,ab

#9 #7 or #8

#10 (Cohort Studies):ti,ab,kw OR (Case-Control Studies):ti,ab,kw OR (Cross-Sectional Studies):ti,ab,kw OR (Longitudinal Studies):ti,ab,kw OR (Follow-Up Studies):ti,ab,kw OR (Prospective Studies):ti,ab,kw OR (Retrospective Studies):ti,ab,kw OR (cohort):ti,ab,kw OR (longitudinal):ti,ab,kw OR (prospective):ti,ab,kw OR (retrospective):ti,ab,kw

#11 #3 and #6 and #9 and #10

**Inclusion and exclusion criteria**

Inclusion criteria: Studies were prospective or retrospective cohort studies based on original data; Data on the risk factors for ESRD reported as risk ratios (RRs) and 95% confidence intervals (95% CIs); Type 2 diabetic patients with eGFR>15 ml min^-1^1.73 m^-2^and did not received kidney replacement therapy (dialysis or transplantation). Exclusion criteria: reviews, commentaries and editorials, irretrievable articles, animal studies and other studies from which data could not be extracted.

**Definition**

Type 2 diabetes was defined as fasting plasma glucose (FPG) of ≥ 7.0 mmol/L, 2-h plasma glucose (2-h PG) value of ≥11.1 mmol/L during a 75 g oral glucose tolerance test (OGTT), glycated hemoglobin A1c (HbA1c) of ≥6.5% (48mmol/mol), use of glucose-lowering drugs, self-reported diabetes, or using administrative data coding algorithms^[3,4]^.The end-point (ESRD) was defined as eGFR<15ml min-11.73 m^-2^ or received kidney replacement therapy(dialysis or transplantation)^[5]^.

**Outcome**

Initiation of ESRD.

**Data extraction and quality assessment**

These meaningful data was extracted to Table 1 by two researchers (QY-R, D-C) independently, including first author, published year, country, details of the study design, sample size, age, DM duration, follow-up, risk factors and RR with 95% CI in each group. We assessed the quality of cohorts using the Newcastle-Ottawa scales. (The NOS contains eight entries and divides into three dimensions: selection, comparability, and outcome). The NOS score ranged from 0 to 9. Score of included articles should be 6 or more, The higher the score, the higher the quality of articles. (**Shown in Supplementary Table 2**)

**Statistical analysis**

The statistical analysis of the data was performed using RevMan (version 5.3.3; The Cochrane Collaboration) and STATA software, version 14.0 (StataCorp, College Station, TX). The results are presented as the risk ratios (RRs) with a 95% confidence interval (95%CI), and a P-value <0.05 was considered statistically significant unless otherwise specified. In addition, heterogeneity was quantified using the Q test and I^2^ statistics. When the heterogeneity test indicated no significant (P>0.1and I^2^<50%), a fixed-effects model was applied; otherwise, a random-effects model was used.

**Results**

We found 1044 articles from Pubmed, 401 from Corchrane Library, 3489 from Embase.804 duplicate articles were excluded.521 articles such as reviews and animal experiments were excluded.3549 articles were excluded after carefully reviewing the titles and abstracts. Of the 60 articles that underwent full-text evaluation, 15 articles met our inclusion criteria ^[6,20]^.

**Characteristics and quality of included studie**s

Of the 15 included studies,12 were prospective cohorts and 3 were retrospective cohorts. There were 1,167,317 patients with diabetes and 13,187 incident ESRD cases were observed during follow-up, and the estimated incidence of ESRD in type 2 diabetic patients was 1.1%. The duration of follow-up ranged from 2.8 to 25 years, the age of the study participants was between 20 to 80.55% of the participants were from Asia while 45% were from America and Europe. The characteristics of all these 15 cohorts were shown in Supplementary Table 1. According to the Newcastle-Ottawa scales, all of the studies had a quality score over 7, which indicated high quality (**Shown in Supplementary Table 2**)

**Risk factors of ESRD in type 2 diabetic patients**

There were 13 risk factors available from the included cohorts, including age, sex, body mass index (BMI), smoking, diabetes mellitus (DM) duration, hypertension, hemoglobin A1c (HbA1c), fasting plasma glucose (FBG), albuminuria, estimated glomerular filtration rate (eGFR), urine albumin: creatinine ratio (UACR), triglyceride (TG), total cholesterol (TC) (Shown in **Supplementary Table 3**).

Of the 8 risk factors for meta-analysis, there were age, sex, smoking, DM duration, systolic blood pressure (SBP), HbA1c, eGFR, and TG with pooled RRs of 1.11.1.53,1.33,1.02,1.01,1.10,0.97and 1.75, respectively. Considering the feasibility of clinical practice, we chose the results from subgroup analysis or sensitivity analysis which were more reasonable. Age increment by 5-10 years old (RR 1.11; 95%CI 1.01-1.21; *P*<0.01). Male (RR 1.53; 95%CI 1.4-1.67; *P*<0.01). Smoking (RR 1.33; 95%CI 1.15-1.53; *P*<0.01);diabetes duration increment by 1 year (RR 1.02; 95%CI 1.01-1.03; *P*<0.01);SBP increment by 1 mmHg (RR 1.01; 95%CI 1.00-1.01; *P*<0.01);HbA1c increment by 1% (RR 1.10; 95%CI 1.08-1.12; *P*<0.01); eGFR increment by 1 min^-1^1,73m^-2^(RR 0.97; 95%CI 0.95-0.99; *P*<0.01);TG increment by 1 mmol/L (RR 1.75; 95%CI 1.34-2.29; *P*<0.01).The details of these risk factors in the involving studies are shown in **Supplementary Figure3-10.**

**Supplementary Table 1.** **Baseline characteristics and risk factors of the 15 cohort studies**

| First author | Year | Country | Source  of cohort | Study design | Sample size  (male/%) | Age  (years) | DM duration  (years) | Follow-up  (years) | Risk  factors |
| --- | --- | --- | --- | --- | --- | --- | --- | --- | --- |
| Linda | 1989 | America | Mayo Clinic and the Rochester Epidemiology Project | Prospec-tive | 891/44.1 | NR | NR | 25 | (1),(2),(4),  (5),(6),(8),  (10), |
| Lee | 1994 | America | Indian Hospitals of U.S. Public Health Service | Retrospective | 378/37.4 | 52.1±11.3 | 7.0±6.4 | 10.2 | (1),(5),(6),  (8) |
| Bruno | 2003 | Italy | Italy type 2 daibetic patients | Prospec-tive | 614/43.6 | 68.1±10.5 | 10.7±6.9 | 6.7 | (2),(3),(4),  (6),(9),(10),(13) |
| Keane | 2003 | 28 countries | Angiotensin Ⅱ Antagonist Losartan(RENALL) Study | Prospec-tive | 956/63.2 | 60.3±7.3 | NR | 3.4 | (12),(13) |
| Yang | 2006 | China | Hong Kong Diabetes Registry | Prospec-tive | 1894/42.7 | NR | 2-11 | 2.9 | (5),(6),(11),(12) |
| Hauteclocque | 2014 | France | SURDIAGENE study | Prospec-tive | 486/42.4 | 65±11 | 14.6±10.4 | 5.7 | (1),(2),(5),  (6),(7),(11),(12) |
| Chang | 2015 | China | Taiwan National Health Insurance Research Database | Prospec-tive | 291745/52.1 | 56.5±11.6 | NR | 5.7 | (1),(2),(6), |
| Meda E | 2013 | Pima Indians | A study of diabetes and its complications | Prospec-tive | 79/34 | 42.8±10.5 | 12.3±6.2 | 10.7 | (1),(2),(7).  (11),(12) |
| Gu | 2016 | China | Staged Diabetes Targeting Management study | Prospec-tive | 1055/64.1 | 64.2±11.9 | 7.0-9.0 | 4.1 | (5),(7),(11) |
| Li | 2016 | China | National Taiwan University Hospital | Prospec-tive | 267/44.2 | 61.7±9.7 | 8(3-14) | 12.4 | (3),(4),(5),(6),(7),(9),(10),(11) |
| Eric Yuk | 2017 | China | Chinese Type 2 diabetes patients | Retrospective | 36289/46.7 | 61.4±10.0 | 6.8±6.1 | 5 | (1),(3),(4),  (5),(6),(7),  (11) |
| Bancha | 2019 | Royal | Department of Internal Medicine | Prospec-tive | 139/54.1 | 66.2±10.6 | 10(5-7) | 3 | (1),(3),(11),(12),(13) |
| Patrik | 2019 | Finland | Diabetes in Finland database | Prospec-tive | 213310/49.4 | 20-79 | NR | 24 | (1),(2) |
| Roland | 2014 | Canada | Healthcare system  administrative data | Retrospective | 1355/51.3 | NR | 11.2±5.5 | 10 | (5) |
| Dong | 2021 | China | Chinese Type 2 diabetes patients | Prospec-tive | 63438/44.8 | 65.6±11.9 | 7.9±6.5 | 9.8 | (1),(5),(6),(7),(11) |

Note: NR=Not reported (1).Age (2).Sex (3)BMI (4).Smoker (5).DM duration (6).Hypertension (7).HbA1c (8).FBG (9).TC (10).Albuminuria (11).eGFR (12).UACR (13).TG

| **First author/year** | **Selection** | | | | **Compara-bility** | **Outcome** | | | **Total**  **scores** |
| --- | --- | --- | --- | --- | --- | --- | --- | --- | --- |
|  | Representa-tiveness of the exposed cohort | Selection of the non- exposed cohort | Ascertain-ment of exposure | Demonstration  that outcome of interest was not present at start of study | Comparability of cohorts on the basis of the design or analysis | Assess-  ment of outcome | Was follow-up long enough for outcomes to occur | Adequacy of follow up of cohorts |  |
| Linda/1989 | ☆ | ☆ | ☆ | ☆ | ☆☆ | ☆ | ☆ | ☆ | 9 |
| Lee/1994 | ☆ | ☆ | ☆ | ☆ | ☆☆ | ☆ | ☆ | ☆ | 9 |
| Bruno/2003 | ☆ | ☆ | ☆ | ☆ | ☆☆ | - | ☆ | ☆ | 8 |
| Keane/2003 | ☆ | ☆ | ☆ | ☆ | ☆☆ | ☆ | ☆ | - | 8 |
| Yang/2006 | ☆ | - | ☆ | ☆ | ☆☆ | ☆ | ☆ | ☆ | 8 |
| Hauteclocque/2014 | ☆ | ☆ | ☆ | ☆ | ☆☆ | ☆ | ☆ | ☆ | 9 |
| Chang/2015 | ☆ | ☆ | ☆ | ☆ | ☆☆ | ☆ | ☆ | ☆ | 9 |
| Meda E/2013 | ☆ | ☆ | - | ☆ | ☆☆ | - | ☆ | ☆ | 7 |
| Gu/2016 | ☆ | ☆ | ☆ | ☆ | ☆☆ | ☆ | ☆ | ☆ | 9 |
| Li/2016 | ☆ | ☆ | ☆ | ☆ | ☆☆ | ☆ | ☆ | ☆ | 9 |
| Eric Yuk/2017 | ☆ | ☆ | ☆ | ☆ | ☆☆ | ☆ | ☆ | ☆ | 9 |
| Bancha/2019 | ☆ | ☆ | ☆ | ☆ | ☆☆ | ☆ | ☆ | ☆ | 9 |
| Patrik/2019 | ☆ | ☆ | ☆ | ☆ | ☆☆ | ☆ | ☆ | ☆ | 9 |
| Roland/2014 | ☆ | ☆ | - | ☆ | ☆☆ | - | ☆ | ☆ | 7 |
| Dong/2021 | ☆ | ☆ | ☆ | ☆ | ☆☆ | ☆ | ☆ | ☆ | 9 |

**Supplementary Table 2. Newcastle-Ottawa Quality Assessment Scale of the 15 cohort studies.**

**Supplementary Table 3**. **13 risk factors include in the systematic review and meta-analysis.**

| Risk factors | First author/Year | Sample size | No. of ESRD | Definition of risk factor | RR/HR | 95%CI | *P* value |
| --- | --- | --- | --- | --- | --- | --- | --- |
| Age | Linda/1989 | 2022 | 28 | Increment by 10 years | 1.2 | 0.9-1.7 | >0.05 |
| Age | Lee/1994 | 912 | 146 | Increment by 1 year | 1.06 | 1.03-1.09 | <0.01 |
| Age | Chang/2015 | 559864 | 1695 | 56-65 (vs.45-55) year | 0.95 | 0.84-1.08 | >0.05 |
| Age | Chang/2015 | 559864 | 1695 | >65 (vs.45-55) year | 0.89 | 0.78-1.02 | >0.05 |
| Age | Hauteclocque/2014 | 1146 | 30 | Increment by 1 year | 1.02 | 1.01-1.04 | <0.01 |
| Age | Meda E/2015 | 193 | 62 | Increment by 5 years | 1.1 | 1.0-1.2 | <0.05 |
| Age | Bancha/2019 | 257 | 67 | Increment by 1 year | 0.98 | 0.95-1.01 | 0.224 |
| Age | Eric Yuk/2017 | 36289 | 91 | Increment by 1 year | 1.06 | 1.05-1.08 | <0.01 |
| Age | Eric Yuk/2017 | 41347 | 70 | Increment by 1 year | 1.03 | 1.02-1.05 | <0.01 |
| Age | Dong/2021 | 52107 | 4609 | Increment by 1 year | 1.03 | 1.02-1.03 | <0.01 |
| Age | Dong/2021 | 42143 | 3887 | Increment by 1 year | 1.02 | 1.01-1.02 | <0.01 |
| Age | Patrik/2019 | 421429 | 1516 | 50-59 (vs.40-49) year | 0.82 | 0.71-0.94 | <0.05 |
| Age | Patrik/2019 | 421429 | 1516 | 60-69 (vs.40-49) year | 0.70 | 0.60-0.81 | <0.05 |
| Age | Patrik/2019 | 421429 | 1516 | 70-79 (vs.40-49) year | 0.45 | 0.37-0.54 | <0.05 |
| Age | Patrik/2019 | 421429 | 1516 | >80 (vs.40-49) year | 0.09 | 0.05-0.14 | <0.05 |
| Sex | Linda/1989 | 2022 | 28 | Male | 2.8 | 1.2-6.9 | <0.05 |
| Sex | Bruno/2003 | 1408 | 82 | Male | 1.28 | 0.79-2.06 | 0.31 |
| Sex | Hauteclocque/2014 | 1146 | 30 | Male | 1.09 | 0.44-2.71 | 0.8 |
| Sex | Hauteclocque/2014 | 1146 | 30 | Male | 1.33 | 1.02-1.76 | 0.04 |
| Sex | Chang/2015 | 559864 | 1695 | Female | 0.64 | 0.58-0.71 | <0.05 |
| Sex | Meda E/2015 | 193 | 62 | Male | 1.6 | 0.9-3.0 | >0.05 |
| Sex | Meda E/2015 | 193 | 62 | Male | 1.7 | 0.9-3.1 | >0.05 |
| Sex | Patrik/2019 | 421429 | 1516 | Male | 1.93 | 1.72-2.16 | <0.05 |
| BMI | Bruno/2003 | 1408 | 82 | 24.2-26.7(vs.<24.2) kg/m^2^ | 1.91 | 0.88-4.12 | >0.05 |
| BMI | Bruno/2003 | 1408 | 82 | 26.8-30.0(vs.<24.2) kg/m^2^ | 2.51 | 1.00-4.61 | <0.05 |
| BMI | Bruno/2003 | 1408 | 82 | >30.0(vs.<24.2) kg/m^2^ | 2.76 | 1.30-5.83 | <0.05 |
| BMI | Eric Yuk/2017 | 41347 | 70 | Increment by 1 kg/m^2^ | 0.85 | 0.77-0.95 | <0.01 |
| BMI | Li/2016 | 604 | 22 | 24-27(vs.<24) kg/m^2^ | 3.74 | 0.88-16.6 | >0.05 |
| BMI | Li/2016 | 604 | 22 | ≥27(vs.<24) kg/m^2^ | 0.96 | 0.25-3.76 | >0.05 |
| BMI | Bancha/2019 | 257 | 67 | Increment by 1 kg/m^2^ | 1.01 | 0.95-1.08 | 0.78 |
| Smoker | Linda/1989 | 2022 | 28 | Smoker | 2.7 | 1.2-6.0 | <0.05 |
| Smoker | Bruno/2003 | 1408 | 82 | Smoker | 1.21 | 0.55-2.63 | >0.05 |
| Smoker | Eric Yuk/2017 | 36289 | 91 | Smoker | 1.29 | 1.11-1.5 | <0.01 |
| Smoker | Li/2016 | 604 | 22 | Smoker | 2.03 | 0.65-6.37 | >0.05 |
| diabetes duration | Linda/1989 | 2022 | 28 | Increment by 10 years | 1.6 | 0.8-3.1 | >0.05 |
| diabetes duration | Lee/1994 | 912 | 146 | Increment by 1 year | 1.05 | 1.01-1.09 | 0.002 |
| diabetes duration | Yang/2006 | 4438 | 159 | Increment by 1 year | 1.06 | 1.02-1.09 | 0.001 |
| diabetes duration | Roland/2014 | 2640 | 30 | Increment by 1 year | 1.11 | 1.04-1.19 | 0.003 |
| diabetes duration | Gu/2016 | 1645 | 37 | Increment by 1 month | 1.004 | 1.00-1.008 | 0.028 |
| diabetes duration | Eric Yuk/2017 | 41347 | 70 | Increment by 1 year | 1.01 | 1.00-1.02 | 0.046 |
| diabetes duration | Dong/2021 | 52107 | 4609 | Increment by 1 year | 1.02 | 1.015-1.026 | <0.01 |
| diabetes duration | Dong/2021 | 52107 | 4609 | Increment by 1 year | 1.016 | 1.009-1.022 | <0.01 |
| diabetes duration | Hauteclocque/2014 | 1146 | 30 | Increment by 1 year | 0.97 | 0.96-0.98 | 0.08 |
| diabetes duration | Li/2016 | 604 | 22 | Increment by 1 year | 1.00 | 0.95-1.06 | >0.05 |
| Hypertension | Linda/1989 | 2022 | 28 | Hypertension | 1.5 | 0.7-3.5 | >0.05 |
| Hypertension | Lee/1994 | 912 | 146 | Hypertension | 1.81 | 1.13-2.91 | 0.01 |
| Hypertension | Hauteclocque/2014 | 1146 | 30 | SBP Increment by 1 mmHg | 1.00 | 0.99-1.01 | 0.6 |
| Hypertension | Bruno/2003 | 1408 | 82 | Hypertension | 6.75 | 1.63-28.01 | 0.009 |
| Hypertension | Bruno/2003 | 1408 | 82 | SBP140–149 (vs.<140) mmHg | 1.68 | 0.66-4.26 | >0.05 |
| Hypertension | Bruno/2003 | 1408 | 82 | SBP150–169 (vs.<140) mmHg | 1.75 | 0.72-4.21 | >0.05 |
| Hypertension | Bruno/2003 | 1408 | 82 | SBP>169 (vs.<140) mmHg | 4.10 | 1.85-9.09 | <0.05 |
| Hypertension | Bruno/2003 | 1408 | 82 | DBP80–85 (vs.<80) mmHg | 1.44 | 0.52-4.03 | >0.05 |
| Hypertension | Bruno/2003 | 1408 | 82 | DBP86–92 (vs.<80) mmHg | 2.05 | 0.75-5.57 | >0.05 |
| Hypertension | Bruno/2003 | 1408 | 82 | DBP>92 (vs.<80) mmHg | 4.08 | 1.53-10.88 | <0.05 |
| Hypertension | Yang/2006 | 4438 | 159 | SBP Increment by 10 mmHg | 1.37 | 1.24-1.51 | <0.01 |
| Hypertension | Chang/2015 | 559864 | 1695 | Increment by 1 mmHg | 2.10 | 1.90-2.31 | <0.05 |
| Hypertension | Eric Yuk/2017 | 36289 | 91 | SBP Increment by 1 mmHg | 1.01 | 1.01-1.02 | <0.01 |
| Hypertension | Eric Yuk/2017 | 41347 | 70 | SBP Increment by 1 mmHg | 1.01 | 1.00-1.01 | 0.025 |
| Hypertension | Eric Yuk/2017 | 36289 | 91 | DBP Increment by 1 mmHg | 0.93 | 0.88-0.98 | 0.009 |
| Hypertension | Eric Yuk/2017 | 41347 | 70 | DBP Increment by 1 mmHg | 0.92 | 0.86-0.98 | 0.007 |
| Hypertension | Dong/2021 | 52107 | 4609 | SBP Increment by 1 mmHg | 1.009 | 1.006-1.011 | <0.01 |
| Hypertension | Dong/2021 | 42143 | 3887 | SBP Increment by 1 mmHg | 1.012 | 1.01-1.015 | <0.01 |
| Hypertension | Dong/2021 | 52107 | 4609 | DBP Increment by 1 mmHg | 0.99 | 0.985-0.994 | <0.01 |
| Hypertension | Dong/2021 | 42143 | 3887 | DBP Increment by 1 mmHg | 0.986 | 0.981-0.991 | <0.01 |
| Hypertension | Li/2016 | 604 | 22 | Hypertension | 1.27 | 0.38-4.26 | >0.05 |
| HbA1c | Meda E/2015 | 193 | 62 | Increment by 1 %(11mmol/mol) | 1.2 | 1.03-1.4 | <0.05 |
| HbA1c | Meda E/2015 | 193 | 62 | Increment by 1 %(11mmol/mol) | 1.2 | 1.04-1.4 | <0.05 |
| HbA1c | Hauteclocque/2014 | 1146 | 30 | Increment by 1 %(11mmol/mol) | 1.12 | 1.03-1.21 | 0.01 |
| HbA1c | Gu/2016 | 1645 | 37 | Increment by 1 %(11mmol/mol) | 1.221 | 1.023-1.457 | 0.027 |
| HbA1c | Eric Yuk/2017 | 36289 | 91 | Increment by 1 %(11mmol/mol) | 0.79 | 0.64-0.98 | 0.036 |
| HbA1c | Eric Yuk/2017 | 41347 | 70 | Increment by 1 %(11mmol/mol) | 0.7 | 0.49-0.99 | 0.046 |
| HbA1c | Dong/2021 | 52107 | 4609 | Increment by 1 %(11mmol/mol) | 1.098 | 1.07-1.128 | <0.01 |
| HbA1c | Dong/2021 | 42143 | 3887 | Increment by 1 %(11mmol/mol) | 1.086 | 1.06-1.114 | <0.01 |
| HbA1c | Li/2016 | 604 | 22 | Increment by 1 %(11mmol/mol) | 1.39 | 1.00-1.95 | >0.05 |
| FBG | Linda/1989 | 2022 | 28 | Increment by 100mg/dL | 2.3 | 1.7-3.0 | <0.05 |
| FBG | Lee/1994 | 912 | 146 | Increment by 100mg/dL | 1.14 | 1.09-1.19 | <0.01 |
| TC | Bruno/2003 | 1408 | 82 | (4.97-5.69) (vs. <4.97) mmol/L | 1.04 | 0.56-1.96 | >0.05 |
| TC | Bruno/2003 | 1408 | 82 | (5.70-6.49) (vs. <4.97) mmol/L | 0.97 | 0.50-1.88 | >0.05 |
| TC | Bruno/2003 | 1408 | 82 | (>6.49) (vs. <4.97) mmol/L | 0.73 | 0.36-1.46 | >0.05 |
| TC | Li/2016 | 604 | 22 | Increment by 1 mmol/L | 1.00 | 0.64-1.56 | >0.05 |
| Albuminuria | Linda/1989 | 2022 | 28 | Albuminuria | 15.8 | 6.7-37.6 | <0.05 |
| Albuminuria | Bruno/2003 | 1408 | 82 | Microalbuminuria | 1.86 | 0.93-3.73 | >0.05 |
|  | Bruno/2003 | 1408 | 82 | Macroalbuminuria | 5.52 | 2.90-10.51 | <0.05 |
| Albuminuria | Li/2016 | 604 | 22 | Albuminuria | 8.30 | 2.09-32.99 | <0.05 |
| eGFR | Yang/2006 | 4438 | 159 | Each 10 ml min^-1^1.73m^-2^ rise | 0.67 | 0.56-0.79 | <0.01 |
| eGFR | Yang/2006 | 4438 | 159 | Each 10 ml min^-1^1.73m^-2^ rise | 0.73 | 0.61-0.88 | <0.01 |
| eGFR | Hauteclocque/2014 | 1146 | 30 | <60 ml min^-1^1.73m^-2^ | 12.31 | 3.60-42.04 | <0.01 |
| eGFR | Meda E/2015 | 193 | 62 | Log2 GFR ( Each 1 ml min^-1^1.73m^-2^ rise ) | 2.3 | 1.5-3.6 | <0.05 |
| eGFR | Meda E/2015 | 193 | 62 | Log2 GFR ( Each 1 ml min^-1^1.73m^-2^ rise ) | 2.1 | 1.4-3.3 | <0.05 |
| eGFR | Li/2016 | 604 | 22 | Each 1 ml min^-1^1.73m^-2^ rise | 0.95 | 0.92-0.98 | <0.01 |
| eGFR | Gu/2016 | 1645 | 37 | Each 1 ml min^-1^1.73m^-2^ rise | 1.012 | 1.001-1.023 | 0.032 |
| eGFR | Eric Yuk/2017 | 36289 | 91 | (60-89) (vs.>90) ml min^-1^1.73m^-2^ | 2.45 | 2.08-2.88 | <0.01 |
| eGFR | Eric Yuk/2017 | 41347 | 70 | (60-89) (vs.>90) ml min^-1^1.73m^-2^ | 2.00 | 0.38-10.51 | 0.412 |
| eGFR | Eric Yuk/2017 | 36289 | 91 | (<60) (vs.>90) ml min^-1^1.73m^-2^ | 8.76 | 7.08-10.94 | <0.01 |
| eGFR | Eric Yuk/2017 | 41347 | 70 | (<60) (vs.>90) ml min^-1^1.73m^-2^ | 112.06 | 23.97-523.76 | <0.01 |
| eGFR | Dong/2021 | 52107 | 4609 | Each 1 ml min^-1^1.73m^-2^ rise | 0.977 | 0.966-0.988 | <0.01 |
| eGFR | Dong/2021 | 52107 | 4609 | Each 1 ml min^-1^1.73m^-2^ rise | 0.978 | 0.967-0.988 | <0.01 |
| eGFR | Dong/2021 | 42143 | 3887 | Each 1 ml min^-1^1.73m^-2^ rise | 0.969 | 0.966-0.971 | <0.01 |
| eGFR | Dong/2021 | 42143 | 3887 | Each 1 ml min^-1^1.73m^-2^ rise | 0.969 | 0.967-0.971 | <0.01 |
| eGFR | Bancha/2019 | 257 | 67 | Each 1 ml min^-1^1.73m^-2^ rise | 1.01 | 0.99-1.02 | 0.218 |
| UACR | Yang/2006 | 4438 | 159 | Log10 UACR (Increment by 1 mg/mmol) | 3.76 | 2.38-5.94 | <0.01 |
| UACR | Yang/2006 | 4438 | 159 | Log10 UACR (Increment by 1 mg/mmol) | 4.28 | 2.66-6.88 | <0.01 |
| UACR | Keane/2003 | 1513 | 686 | Log10 UACR (Increment by 1 mg/g) | 6.2 | 4.4-8.7 | <0.01 |
| UACR | Hauteclocque/2014 | 1146 | 30 | Log10 UACR (Increment by 1 mg/mmol) | 7.39 | 3.84-14.20 | <0.01 |
| UACR | Hauteclocque/2014 | 1146 | 30 | Log10 UACR (Increment by 1 mg/mmol) | 1.97 | 1.65-2.34 | <0.01 |
| UACR | Meda E/2015 | 193 | 62 | Log2 UACR (Increment by 1 mg/g) | 1.5 | 1.3-1.7 | <0.05 |
| UACR | Bancha/2019 | 257 | 67 | Increment by 1 mg/g | 1.01 | 1.01-1.02 | <0.01 |
| TG | Bancha/2019 | 257 | 67 | Increment by 1 mmol/L | 1.03 | 0.28-3.78 | 0.961 |
| TG | Keane/2003 | 1513 | 686 | Increment by 1 mg/dL | 1.7 | 1.2-2.4 | 0.002 |
| TG | Bruno/2003 | 1408 | 82 | (1.06-1.43) (vs.<1.06) mmol/L | 1.95 | 0.91-4.17 | >0.05 |
| TG | Bruno/2003 | 1408 | 82 | (1.44-2.03) (vs.<1.06) mmol/L | 1.37 | 0.61-3.08 | >0.05 |
| TG | Bruno/2003 | 1408 | 82 | (>2.03) (vs.<1.06) mmol/L | 2.6 | 1.26-5.36 | <0.01 |

Note: BMI, body mass index; HbA1c, Hemoglobin A1c; SBP, systolic blood pressure; DBP, diastolic blood pressure; FPG, fasting plasma glucose; TC, total cholesterol; TG, triglyceride; eGFR, estimated glomerular filtration rate; UACR, urine albumin: creatinine ratio;

**Supplementary Table 4：Baseline characteristics of subjects in validation cohort**

| Variables | Total | Development of ESRD | | *P* value |
| --- | --- | --- | --- | --- |
|  |  | No | Yes |  |
| N | 520 | 442 | 78 |  |
| Follow-up(months) | 36.0(26.0,48.0) | 36.7(26.0-48.0) | 38.6(26.0,49.3) | 0.016 |
| Age(years) | 58.8±12.8 | 58.6±12.9 | 60.1±11.9 | 0.439 |
| Male(%) | 313(60.2) | 256(57.9) | 57(73.1) |  |
| Smoker[n (%)] | 250(48.0) | 199(45.0) | 51(65.4) | 0.001 |
| Diabetes duration(years) | 10.6(4.25,17.0) | 10.9(4.0,16.0) | 13.2(8.0,20.0) | < 0.01 |
| SBP(mmHg) | 132.5(122.0,144.0) | 130.3(120.0,140.0) | 157.5(146.5,170.0) | < 0.01 |
| HbA1c(%)  [mmol/mol] | 7.8(7.0,9.5)  [61.7(53.0,80.3)] | 8.5(7.0,9.6)  [69.4(53.0,81.42)] | 7.5(6.1,8.8)  [55.1(43.2,68.4)] | < 0.01 |
| eGFR  (ml min^-1^1.73 m^-2^) | 92.1(76.2,103.4) | 93.1(84.0,105.8) | 63.6(50.3,76.6) | < 0.01 |
| TG(mmol/L) | 1.38(1.0,1.9) | 1.62(1.03,1.91) | 1.93(0.98,2.49) | 0.354 |
| OAD[n(%)] | 235(45.1) | 203(45.9) | 32(41.0) | 0.423 |
| Insulin[n(%)] | 226(43.4) | 196(44.3) | 30(38.4) | 0.334 |
| OAD with Insulin[n(%)] | 232(44.6) | 191(43.2) | 41(52.5) | 0.126 |
| ACEI/ARB[n(%)] | 171(32.9) | 140(31.7) | 31(39.8) | 0.162 |
| Statins[n(%)] | 195(37.5) | 172(38.9) | 23(29.5) | 0.11 |

OAD, Oral antidiabetic drug.

**Supplementary Table 5: The sensitivity, specificity and Youden index of different cutoff risk scores**

| Cut-off  value | Sensitivity | Specificity | Youden  index |
| --- | --- | --- | --- |
| 2 | 100.0% | 0.00% | 0.00% |
| 3 | 100.0% | 0.45% | 0.45% |
| 4 | 100.0% | 0.90% | 0.90% |
| 5 | 100.0% | 2.02% | 2.02% |
| 6 | 100.0% | 3.60% | 3.60% |
| 7 | 100.0% | 5.62% | 5.62% |
| 8 | 100.0% | 9.21% | 9.21% |
| 8.5 | 100.0% | 11.01% | 11.01% |
| 9 | 100.0% | 11.24% | 11.24% |
| 9.5 | 100.0% | 15.73% | 15.73% |
| 10 | 100.0% | 16.63% | 16.63% |
| 10.5 | 97.33% | 22.25% | 19.58% |
| 11 | 96.00% | 22.70% | 18.70% |
| 11.5 | 96.00% | 28.31% | 24.31% |
| 12 | 93.33% | 29.89%% | 23.22% |
| 12.5 | 93.33% | 34.38% | 27.71% |
| 13 | 93.33% | 35.28% | 28.61% |
| 13.5 | 89.33% | 42.25% | 31.58% |
| 14 | 88.0% | 44.27% | 32.27% |
| 14.5 | 88.0% | 50.79% | 38.79% |
| 15 | 88.0% | 53.03% | 41.03% |
| 15.5 | 86.67% | 57.30% | 43.97% |
| 16* | 85.33% | 60.45% | 45.78% |
| 16.5 | 78.67% | 63.15% | 41.82% |
| 17 | 78.67% | 66.52% | 45.19% |
| 17.5 | 70.67% | 70.56% | 41.23% |
| 18 | 69.33% | 73.03% | 42.36% |
| 18.5 | 68.00% | 76.18% | 44.18% |
| 19 | 65.33% | 79.55% | 44.88% |
| 19.5 | 61.33% | 82.70% | 44.03% |
| 20 | 60.00% | 84.49% | 44.49% |
| 20.5 | 57.33% | 85.84% | 43.17% |
| 21 | 54.67% | 89.66% | 44.33% |
| 21.5 | 50.67% | 91.24% | 41.91% |
| 22 | 46.67% | 93.48% | 40.15% |
| 22.5 | 44.00% | 94.16% | 38.16% |
| 23 | 36.00% | 95.73% | 31.73% |
| 23.5 | 33.33% | 95.96% | 29.29% |
| 24 | 29.33% | 96.85% | 26.18% |
| 24.5 | 28.00% | 97.53% | 25.53% |
| 25 | 26.67% | 98.20% | 24.87% |
| 25.5 | 25.33% | 98.43% | 23.76% |
| 26 | 20.00% | 98.88% | 18.88% |
| 26,5 | 18.67% | 99.10% | 17.77% |
| 27 | 13.33% | 99.10% | 12.43% |
| 27.5 | 8.00% | 99.10% | 7.10% |
| 28 | 6.67% | 99.33% | 6.00% |
| 28.5 | 5.33% | 99.33% | 4.66% |
| 29 | 5.33% | 99.55% | 4.88% |
| 29.5 | 2.67% | 99.55% | 2.22% |
| 30.5 | 1.33% | 99.55% | 0.88% |
| 31.5 | 1.33% | 99.78% | 1.11% |
| 32.5 | 1.33% | 100.00% | 1.33% |
| 32.5 | 0.00% | 100.00% | 0.00% |

* Optimal cutoff point

**Supplementary Figure1:** **RR (95% CI) and** **results of heterogeneity test of the risk factors for ESRD.**


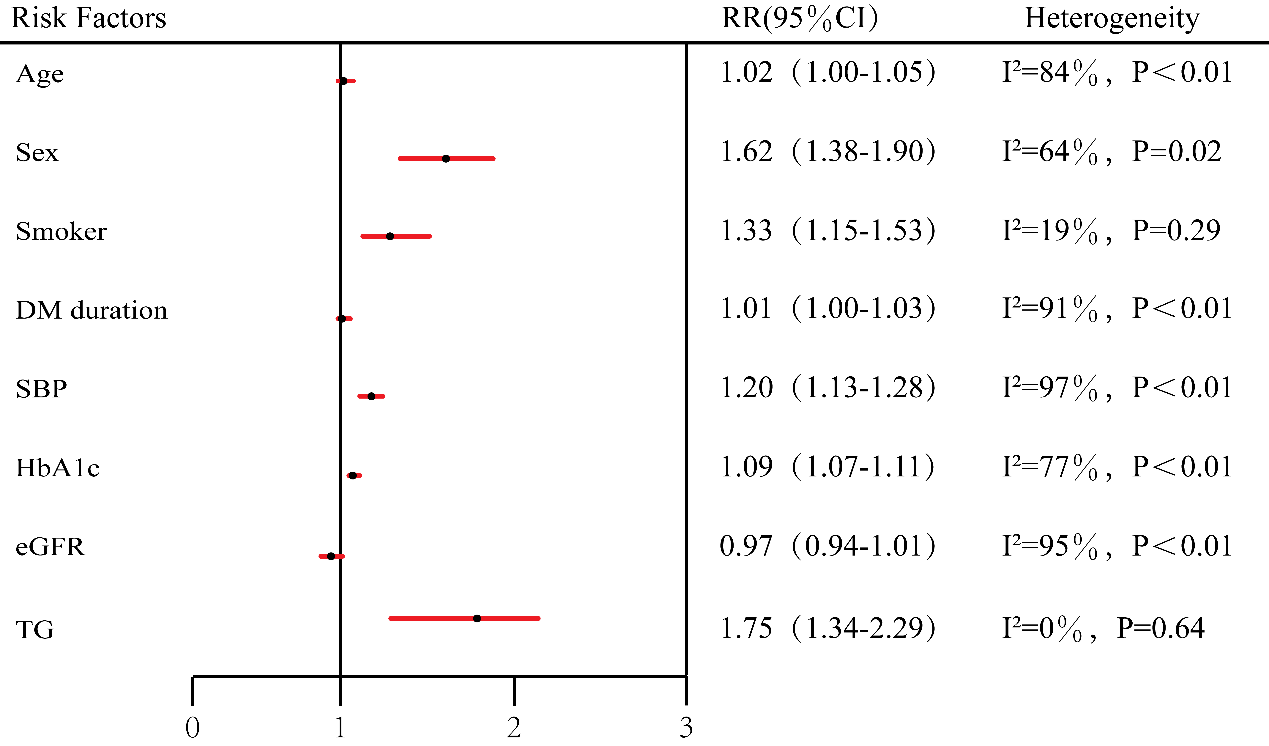


**Supplementary Figure2:** **Subgroup or sensitivity analyses of the risk factors for ESRD.**


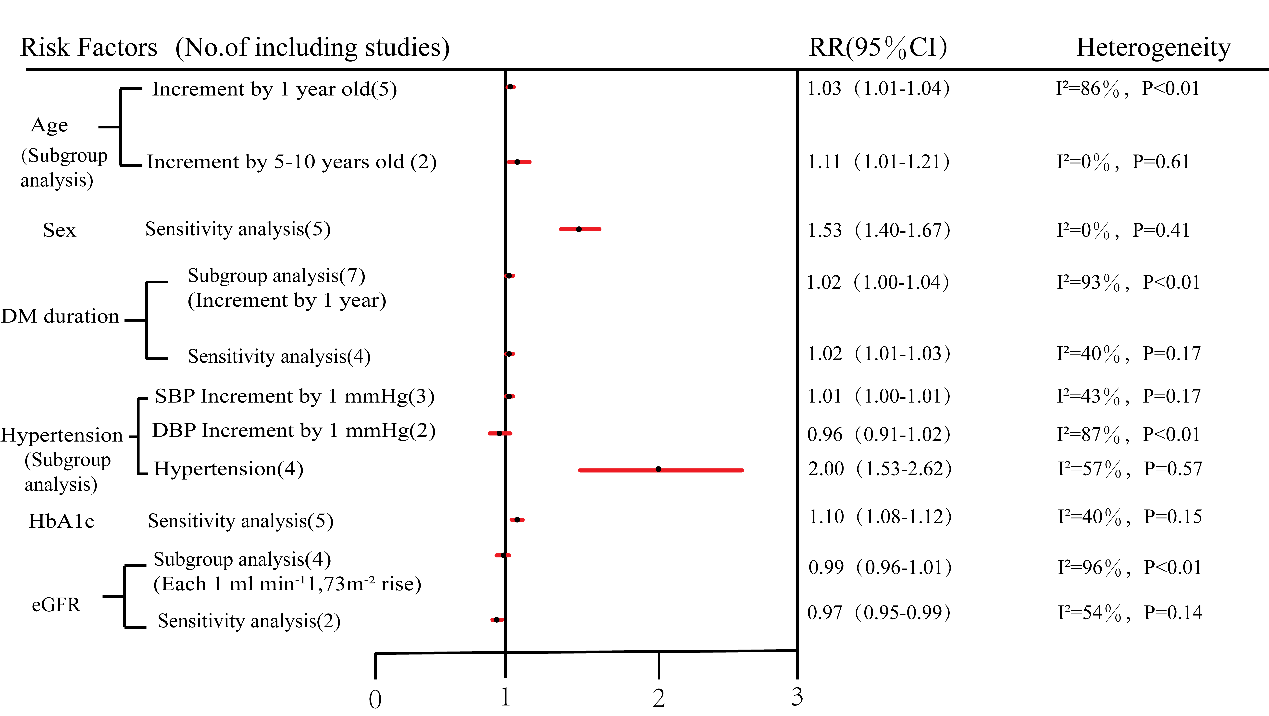


**Supplementary Figure 3.** Age





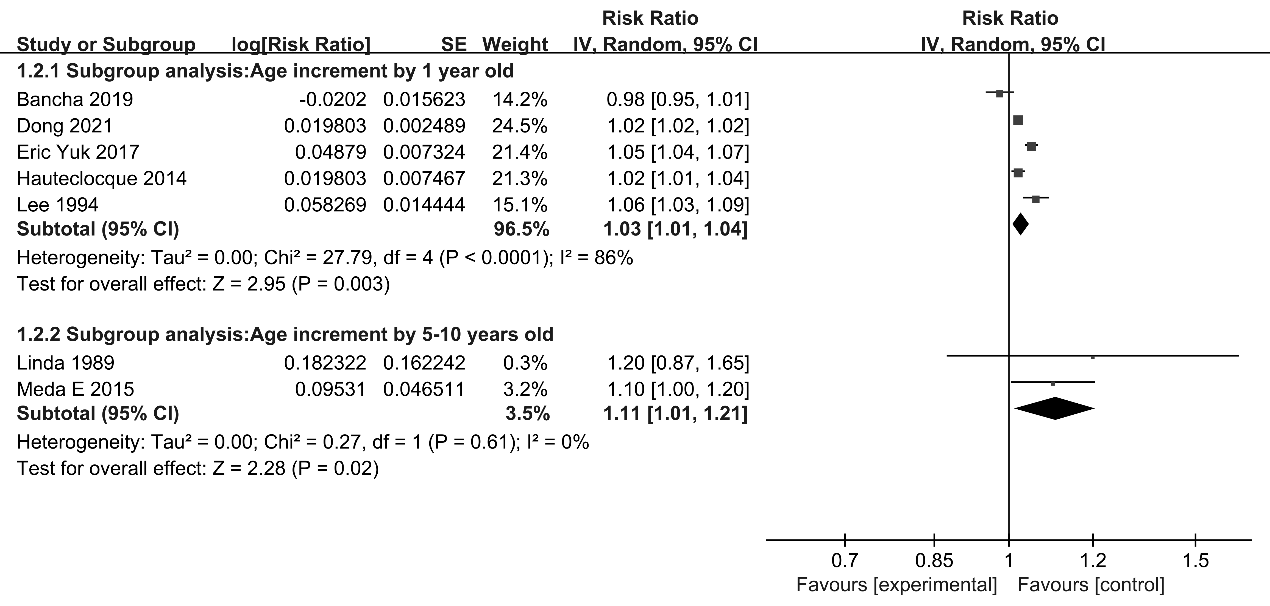


Figure3- a. Association of age with ESRD; b. Subgroup analysis of association of age with ESRD

**Supplementary Figure 4. Sex**



a


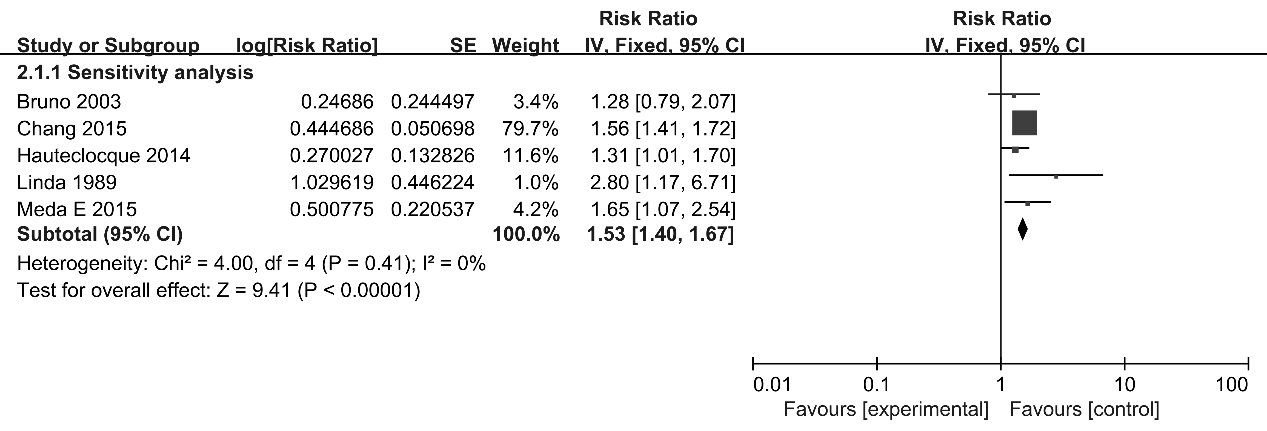
b

Figure 4-a. Association of sex with ESRD; b. Sensitivity analysis of association of sex with ESRD

**Supplementary Figure 5. Smoking**





Figure5. Association of smoking with ESRD

**Supplementary Figure6. DM duration**

a


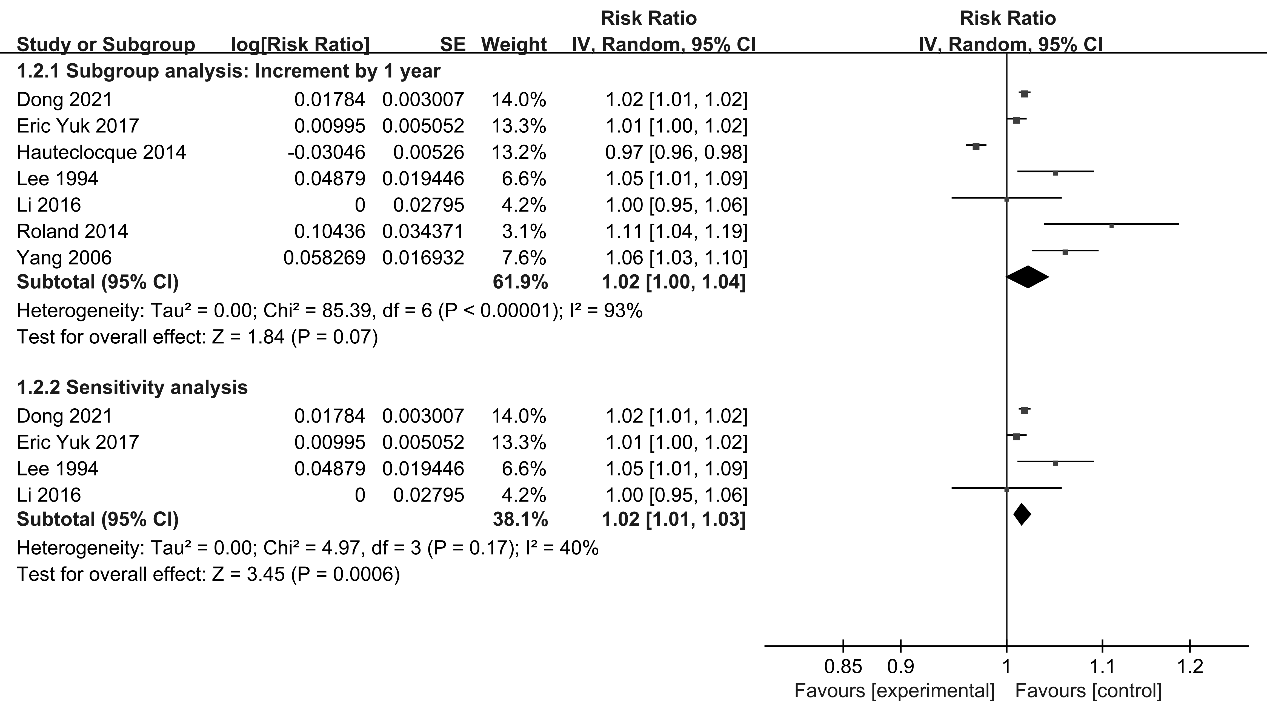

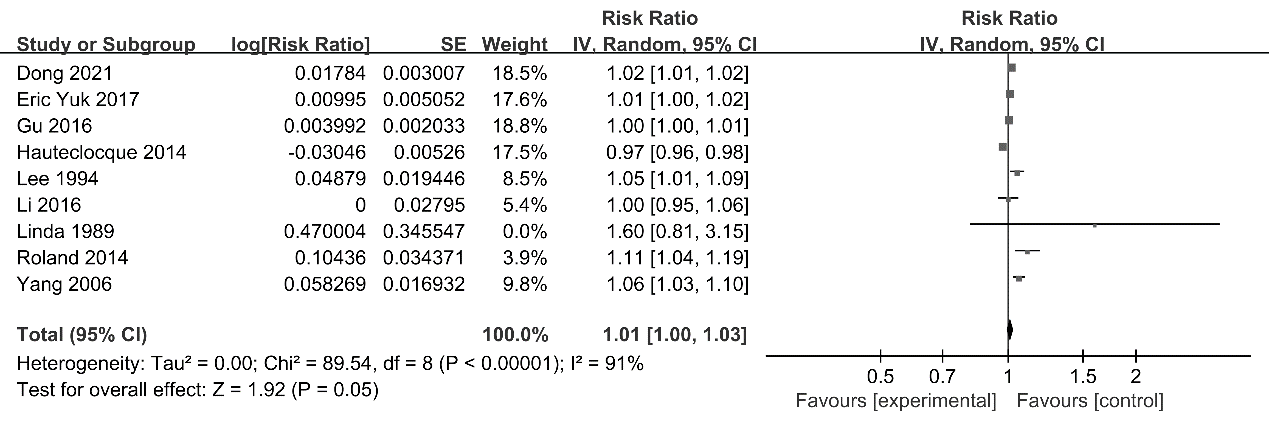


b

Figure 6- a. Association of DM duration with ESRD; b. Sensitivity analysis and subgroup analysis of association of DM duration with ESRD

**Supplementary Figure 7. Hypertension**



a


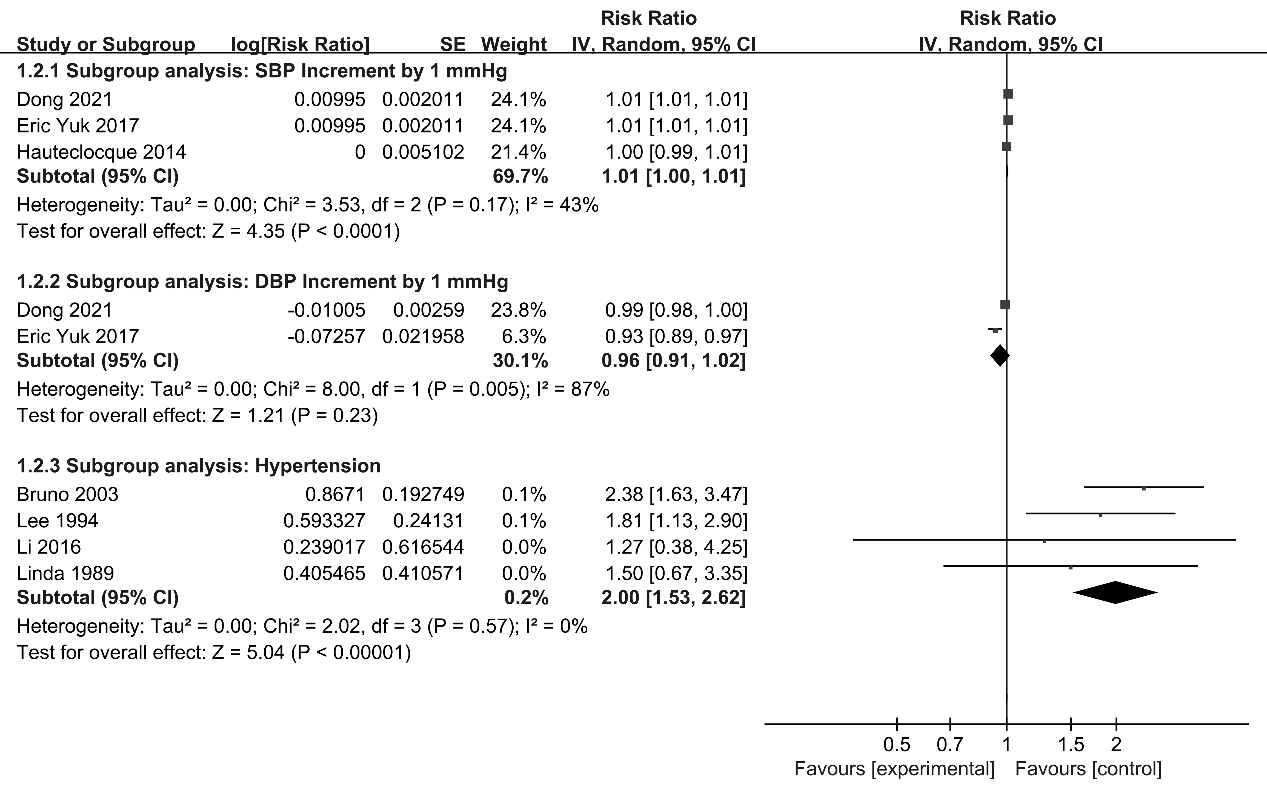
b

Figure7-a. Association of hypertension with ESRD; b. Subgroup analysis of association of hypertension with ESRD

**Supplementary Figure 8. HbA1c**


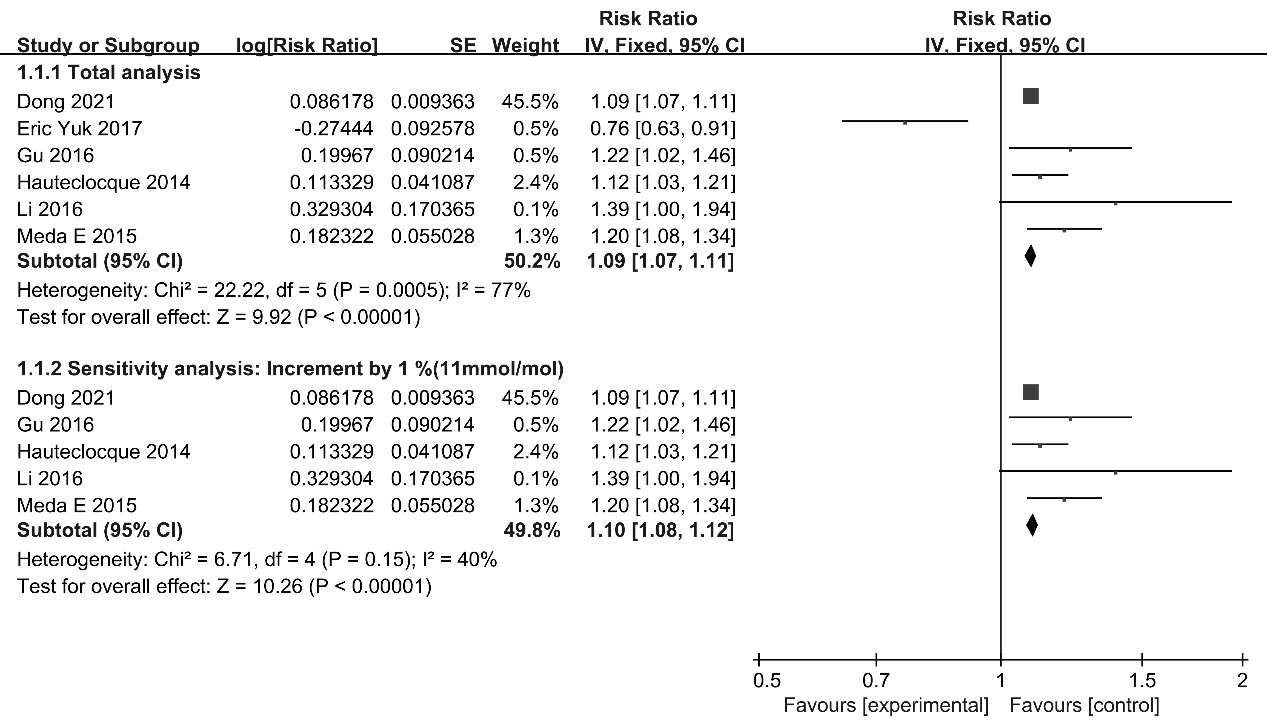


Figure8. Association of HbA1c with ESRD; Sensitivity analysis of association of HbA1c with ESRD

**Supplementary Figure 9. eGFR**



a


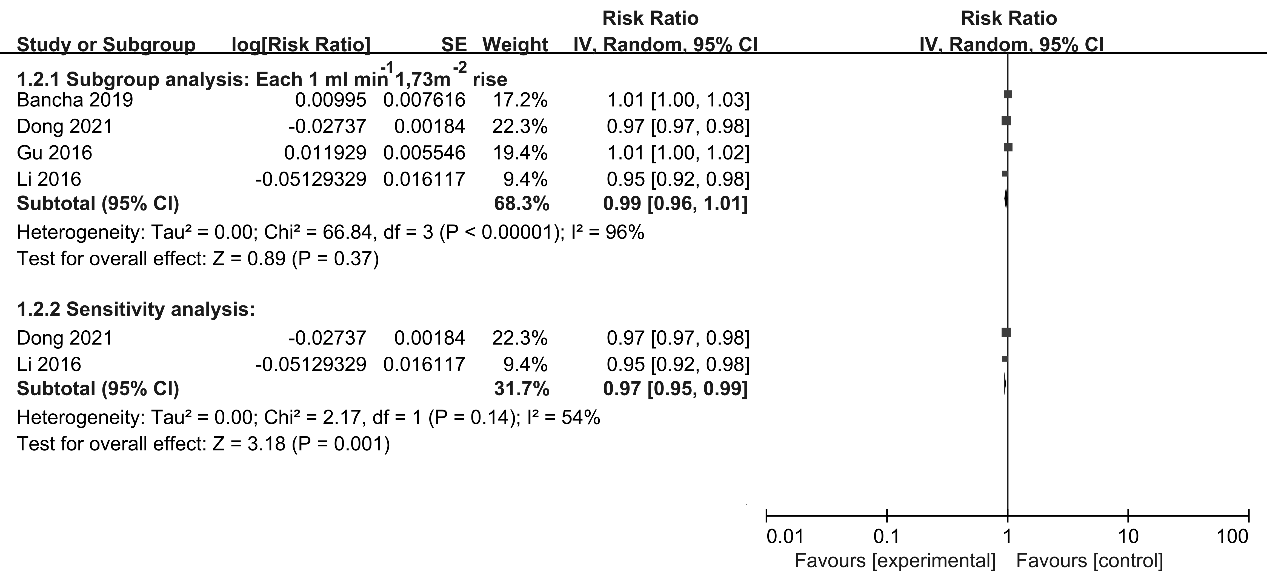
b

Figure9-a. Association of eGFR with ESRD; b. Subgroup and sensitivity analysis of association of eGFR with ESRD

**Supplementary Figure 10. TG**





Figure 10. Association of TG with ESRD

Reference

[1] Shamseer L, Moher D, Clarke M, et al. Preferred reporting items for systematic review and meta-analysis protocols (PRISMA-P) 2015: elaboration and explanation[J]. Bmj, 2015, 350: g7647.

[2] Stroup D F, Berlin J A, Morton S C, et al. Meta-analysis of observational studies in epidemiology: a proposal for reporting. Meta-analysis Of Observational Studies in Epidemiology (MOOSE) group[J]. Jama, 2000, 283(15): 2008-12.

[3] Alberti K G, Zimmet P Z. Definition, diagnosis and classification of diabetes mellitus and its complications. Part 1: diagnosis and classification of diabetes mellitus provisional report of a WHO consultation[J]. Diabet Med, 1998, 15(7): 539-53.

[4] 2. Classification and Diagnosis of Diabetes: Standards of Medical Care in Diabetes-2019[J]. Diabetes Care, 2019, 42(Suppl 1): S13-s28.

[5] KDOQI Clinical Practice Guideline for Diabetes and CKD: 2012 Update[J]. Am J Kidney Dis, 2012, 60(5): 850-86.

[6] Humphrey L L, Ballard D J, Frohnert P P, et al. Chronic renal failure in non-insulin-dependent diabetes mellitus. A population-based study in Rochester, Minnesota[J]. Ann Intern Med, 1989, 111(10): 788-96.

[7] Lee E T, Lee V S, Lu M, et al. Incidence of renal failure in NIDDM. The Oklahoma Indian Diabetes Study[J]. Diabetes, 1994, 43(4): 572-9.

[8] Bruno G, Biggeri A, Merletti F, et al. Low incidence of end-stage renal disease and chronic renal failure in type 2 diabetes: a 10-year prospective study[J]. Diabetes Care, 2003, 26(8): 2353-8.

[9] Keane W F, Brenner B M, De Zeeuw D, et al. The risk of developing end-stage renal disease in patients with type 2 diabetes and nephropathy: the RENAAL study[J]. Kidney Int, 2003, 63(4): 1499-507.

[10] Yang X L, So W Y, Kong A P, et al. End-stage renal disease risk equations for Hong Kong Chinese patients with type 2 diabetes: Hong Kong Diabetes Registry[J]. Diabetologia, 2006, 49(10): 2299-308.

[11] De Hauteclocque A, Ragot S, Slaoui Y, et al. The influence of sex on renal function decline in people with Type 2 diabetes[J]. Diabet Med, 2014, 31(9): 1121-8.

[12] Chang P Y, Chien L N, Lin Y F, et al. Nonadherence of Oral Antihyperglycemic Medication Will Increase Risk of End-Stage Renal Disease[J]. Medicine (Baltimore), 2015, 94(47): e2051.

[13] Pavkov M E, Nelson R G, Knowler W C, et al. Elevation of circulating TNF receptors 1 and 2 increases the risk of end-stage renal disease in American Indians with type 2 diabetes[J]. Kidney Int, 2015, 87(4): 812-9.

[14] Gu L, Lou Q, Wu H, et al. Lack of association between anemia and renal disease progression in Chinese patients with type 2 diabetes[J]. J Diabetes Investig, 2016, 7(1): 42-7.

[15] Li H Y, Lin H A, Nien F J, et al. Serum Vascular Adhesion Protein-1 Predicts End-Stage Renal Disease in Patients with Type 2 Diabetes[J]. PLoS One, 2016, 11(2): e0147981.

[16] Wan E Y F, Fong D Y T, Fung C S C, et al. Prediction of new onset of end stage renal disease in Chinese patients with type 2 diabetes mellitus - a population-based retrospective cohort study[J]. BMC Nephrol, 2017, 18(1): 257.

[17] Satirapoj B, Pooluea P, Nata N, et al. Urinary biomarkers of tubular injury to predict renal progression and end stage renal disease in type 2 diabetes mellitus with advanced nephropathy: A prospective cohort study[J]. J Diabetes Complications, 2019, 33(9): 675-681.

[18] Finne P, Groop P H, Arffman M, et al. Cumulative Risk of End-Stage Renal Disease Among Patients With Type 2 Diabetes: A Nationwide Inception Cohort Study[J]. Diabetes Care, 2019, 42(4): 539-544.

[19] Dyck R F, Jiang Y, Osgood N D. The long-term risks of end stage renal disease and mortality among First Nations and non-First Nations people with youth-onset diabetes[J]. Can J Diabetes, 2014, 38(4): 237-43.

[20] Dong W, Wan E Y F, Fong D Y T, et al. Prediction models and nomograms for 10-year risk of end-stage renal disease in Chinese type 2 diabetes mellitus patients in primary care[J]. Diabetes Obes Metab, 2021, 23(4): 897-909.
